# Biomolecular condensate microstructure is invariant to sequence-encoded molecular and macroscopic properties

**DOI:** 10.1039/d5sm00740b

**Published:** 2025-09-23

**Authors:** Daniel Tan, Dilimulati Aierken, Pablo L. Garcia, Jerelle A. Joseph

**Affiliations:** a Department of Chemical and Biological Engineering, Princeton University Princeton NJ 08544 USA jerellejoseph@princeton.edu; b Omenn-Darling Bioengineering Institute, Princeton University Princeton NJ 08544 USA

## Abstract

Biomolecular condensates, including those formed by prion-like low complexity domains (LCDs) of proteins, are maintained by networks of molecular interactions. Such collective interactions give rise to the rich array of material behaviors underlying condensate function. Previous work has uncovered distinct LCD conformations in condensates *versus* dilute phases, and recently, single-component LCD condensates have been predicted to exhibit microstructures with “small-world” networks—where molecular nodes are highly clustered and connected *via* short pathlengths. However, a framework linking single-molecule properties, condensate microstructure, and macroscopic material properties remains elusive. Here, we combine molecular simulation and graph-theoretic analysis to reveal that small-world microstructures are inherent properties of LCD-like polymers, whose sequence features impact both molecule-scale conformations and droplet-scale material properties while maintaining a stable network structure. Using a residue-resolution coarse-grained model, we probe condensates comprising naturally-occuring LCD sequences and generalize our findings by varying composition and patterning in binary sequences of hydrophobic and polar residues. We show that non-blocky sequences, including a hydrophobic homopolymer, form condensates with small-world internal networks featuring “hubs”—molecules responsible for global connectivity—and “cliques”, molecular clusters bound by persistent short-ranged associations. Cliques localize near interfaces without a secondary phase transition, suggesting a role in mediating molecular partitioning and condensate aging by tuning interfacial material properties. Moreover, we discover that network small-worldness and droplet surface tension are consequences of sequence length and hydrophobicity. We also track single-molecule structure and dynamics inside condensates, revealing that internal heterogeneity at the single-molecule level is systematically encoded by network topology. Collectively, our work establishes multiscale structure–property relationships in LCD condensates, elucidating general organizing principles of the condensate microstructure that persist with sequence-driven changes in molecular behaviors and material properties.

## Abstract 2

Coarse-grained simulations reveal that the internal organization of biomolecular condensates is robust to diverse arrays of sequence-encoded molecular and macroscopic properties.

## Graphical abstract

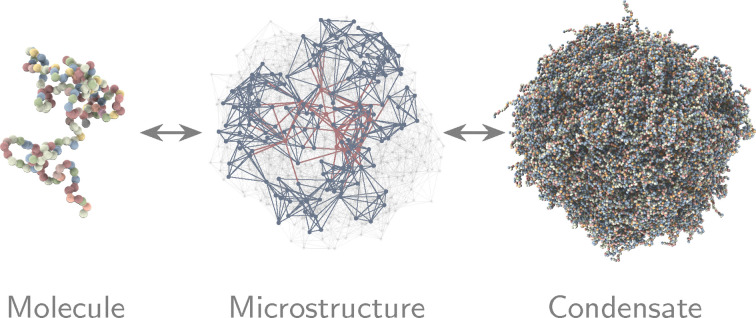

Biomolecular condensates are membraneless organelles inside living cells that exhibit a wide range of material behaviors and functions. Phase separation is a leading mechanism that accounts for condensate formation.^1–4^ In this framework, proteins and nucleic acids minimize free energy by demixing from the cytosol or nucleoplasm, leading to two or more distinct liquid phases.^5,6^ Unlike simple liquids, evidence suggests that diverse sets of specific and nonspecific interactions between biomolecules yield condensates with nontrivial internal architectures—*i.e.*, transient percolated networks.^7–12^ This network microstructure is thought to relate to molecular conformations in the dense phase, as well as to the material properties of condensates.^11,13–15^ Many experiments and simulations have revealed how molecular properties change in condensates compared to dilute phases.^10,16–22^ Recent experiments have also demonstrated that condensates display a diverse array of viscoelastic behaviors,^11,23–30^ and further, that condensate viscoelasticity evolves over time—leading to dynamically arrested states associated with pathological condensate aging.^28,31–33^ While the complex features of condensates have been observed at both single-molecule and droplet scales, we lack a fundamental understanding of the principles that connect molecular-level interactions and conformations to macroscopic material properties and functions. A key missing element in relating phenomena across these length scales is a systematic characterization of the condensate microstructure that accounts for complex internal organization and emergent material properties.

Intrinsically disordered regions (IDRs) are among the key components of proteins involved in intracellular phase separation and condensate formation.^34^ Prion-like low-complexity domains (LCDs) are exemplary instances of IDRs in biomolecular condensation: LCD sequences contain strongly interacting “sticker” residues that drive clustering and phase separation, as well as “spacer” residues interspersed between stickers that modulate solubility and intermolecular interaction strengths.^13,15,35–38^ These sequence architectures enable the formation of complex networks of reversible physical crosslinks underlying condensates.^9,39^ Recent experimental advances and simulation approaches have begun to observe the rich internal organization and heterogeneities associated with condensate microstructures.^10,12,40^ Specifically, Farag *et al.* first leveraged lattice simulation and graphical network analysis to predict the inhomogeneous connectivity of networks underlying LCD condensates, noting a “small-world” graph structure globally connected by a small subset of highly connective “hubs”.^10^ These results were recently supported by experimental studies revealing the inhomogeneous, network-like internal organization of single-component LCD condensates.^12^ Similar network analyses to ref. 10 have been employed to probe molecular networks in two-component condensates,^11^ to determine the effect of temperature, length, and residue composition on networks underlying multicomponent condensates,^41^ and to study the effects of sequence patterning and binding site affinity on percolation and phase separation.^18^ Despite experimental and computational characterization, the principles that govern how single-molecule sequence and structure give rise to condensate microstructures—and how microstructures, in turn, relate to material properties—remain poorly defined.

Here, we address this gap by combining molecular dynamics simulation and graph-theoretic analysis to characterize the microstructures of LCD condensates. Importantly, we show how condensate microstructure gives rise to clustered internal organizations and interfacial properties that persist with large changes in sequence composition, single-molecule behaviors, and macroscopic material properties. We systematically study the interaction networks underlying LCD condensates using a chemically-specific residue-resolution coarse-grained model, Mpipi.^42^ We generalize our findings by designing binary sequences composed of tyrosine (Y) and serine (S) residues to investigate the impact of sequence composition and patterning on network topology. We consistently find that LCDs and non-blocky binary sequences, including a hydrophobic homopolymer, form condensates with small-world microstructures marked by high local clustering of molecules and short global pathlengths. We demonstrate that the small-worldness of LCD microstructures varies systematically with sequence length, while that of the phase-separating binary sequences is independent of sequence hydrophobicity. Moreover, we show that sequence length and hydrophobicity tune droplet surface tension while preserving the small-world characteristics of the network. We further reveal that biomolecules possess two distinct regimes of interactivity in the small-world network. One regime is marked by high global connectivity and expanded conformations (“hubs”), while the other is marked by elevated local crosslinking (“cliques”). By quantifying the spatial and temporal dynamics of the condensate microstructure, we find that cliques display confined local movements and exhibit long lifetimes compared to hubs, whose molecular identities are found to be highly transient. In agreement with previous experimental studies of mesoscale inhomogeneities in condensates, we find that these nanoscale clique clusters consistently form near interfaces without a secondary phase transition, suggesting roles in mediating selective molecular partitioning and condensate aging.^31,32,40^

Our work also demonstrates that the condensate microstructure is shaped by a heterogeneous ensemble of single-molecule conformations in the dense phase. Specifically, we predict power-law-like relationships between network connectivity and single-molecule conformational characteristics including radius of gyration and polymer shape anisotropy, indicating that condensate microstructure can be read out with single-molecule features and *vice versa*. By systematically varying sequence composition and patterning using the binary sequence model, we show that the small-world internal structure is generally not achieved by blocky sequences, which tend to form micelles instead of a distinct liquid phase. However, the relationships between molecular behavior and microstructure are conserved in all phase-separated condensates, spanning a wide range of sequence compositions. Taken together, our work establishes multiscale structure–property relationships of LCD condensates—linking sequence features to molecule-scale behavior and droplet-scale material properties—and provides a conceptual framework for decoding and engineering stable condensates with complex internal architectures robust to a wide range of sequence compositions and material behaviors.

## 1. Results

### 1.1. Characterization of phase behavior and material properties of LCD and YS condensates

**Fig. 1:**
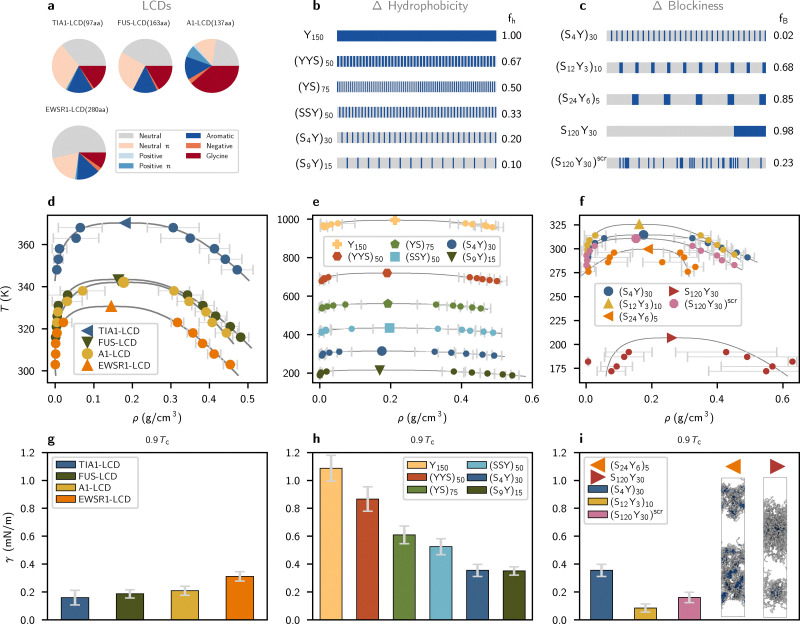
Characterization of phase behavior and material properties of LCD and YS variant sequences. (a) Sequence lengths and compositions of studied LCD sequences. (b) The sequence features of varying hydrophobicity *f*_h_ for YS variant sequences. (c) The sequence features of varying blockiness *f*_B_ for YS variant sequences with fixed hydrophobicity *f*_h_ = 0.20. (d) Phase diagrams for LCD sequences. (e) and (f) Phase diagrams for simulated YS variant sequences. (g) Computed surface tensions for simulated LCD sequences at 0.9*T*_c_. Error bars denote the standard error about the mean. (h) and (i) Computed surface tensions for simulated YS variant sequences at 0.9*T*_c_. As in (g), error bars denote the standard error about the mean.

**Fig. 2:**
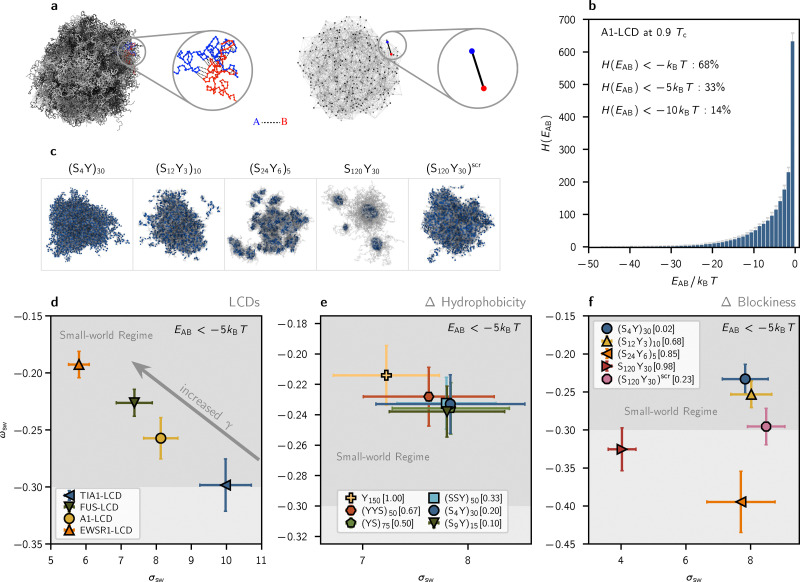
Small-world connectivity in LCD and YS condensates varies with length and is independent of sequence composition. (a) Snapshot of a simulated condensate (left) and its corresponding graph representation (right), with two interacting molecules depicted in the insets. In the graph representation, each molecule is taken as a node, and two nodes are connected with an unweighted, undirected edge when the sum of pairwise monomer interaction energies (*E*_AB_) between them exceeds a threshold based on the thermal energy (*k*_B_*T*). All results and figures in this work use the threshold 5*k*_B_*T*. (b) Histogram depicting the distribution of intermolecular contact energies in a simulation of A1-LCD at 0.9*T*_c_. The 5*k*_B_*T* threshold captures ≈33% of all recorded interactions. (c) Morphologies of selected YS variants are shown, with the two blockiest sequences (S_24_Y_6_)_5_ and S_120_Y_30_ showing micellization with core–shell architecture. (d) Small-world parameters *σ*_sw_ and *ω*_sw_ for single-component LCD condensates. An arrow is overlaid to show that droplet surface tension increases as LCD length increases and the microstructure approaches ideal small-world networking (*ω*_sw_ towards 0). (e) Small-world parameters *σ*_sw_ and *ω*_sw_ for condensates formed by YS sequences with varying hydrophobicity. Small-world statistics appear to be consistent across an order-of-magnitude change in hydrophobic content and surface tension. (f) Small-world parameters *σ*_sw_ and *ω*_sw_ for condensates formed by YS sequences with *f*_h_ = 0.2 and varying patterning. The two blockiest sequences (S_24_Y_6_)_5_ and S_120_Y_30_ form micelles with underlying networks that are distinctly not small-world-like.

To investigate the phase and material properties of biomolecular condensates composed of LCD-like molecules, we perform molecular dynamics (MD) simulations of single-component LCD condensates (left panel in Fig. 2a). Here, we adopt Mpipi^42^—a chemically specific residue-resolution model that has been shown to describe well the phase behavior of disordered proteins. In addition to characterizing natural LCD sequences (*e.g.*, FUS-LCD), we design and simulate ten “YS variant” sequences composed of tyrosine (Y) stickers and serine (S) spacers with varying hydrophobicity and blockiness.^36,43^ Using the YS sequences, we systematically vary the fraction and position of tyrosine residues in the polymers to examine how well our findings for naturally-occurring LCDs extend to sticker–spacer-like polymer architectures (Fig. 1b and c).

We survey biologically relevant condensates by simulating four LCDs known to phase separate under physiological conditions, namely TIA1-LCD, FUS-LCD, hnRNPA1-LCD (“A1-LCD”) and EWSR1-LCD. LCD sequence features are shown graphically in Fig. 1a and exact sequences are given in the Methods. The critical temperatures *T*_c_ of each sequence are estimated using direct coexistence simulations and the data is fitted using the law of coexisting densities and rectilinear diameters.^44,45^ The corresponding phase diagrams are shown in Fig. 1d. Despite being the shortest sequence (*n* = 97 aa), TIA1-LCD is observed to have the highest critical temperature (*T*_c_ = 370 K) of all LCDs, while the longest sequence EWSR1-LCD (*n* = 280 aa) has the lowest predicted critical temperature (*T*_c_ = 327 K). Notably, TIA1-LCD has the highest fraction of π and aromatic residues (*e.g.*, tyrosine); EWSR1-LCD has the lowest fraction of these residues and the highest fraction of neutral and glycine residues. Consistent with previous reports, we observe that strong π–π and cation–π interactions play outsized roles in driving macromolecular phase separation.^36,39,46,47^

All YS sequence variants are constructed with a length of *n* = 150 aa, similar to the average length of the chosen LCD sequences. To study the effect of sequence composition, we varied sequence hydrophobicity (fraction of tyrosine residues, *f*_h_) from *f*_h_ = 0.10 to *f*_h_ = 1.00 over six sequence variants, preserving the near uniform distribution of sticker residues noted for phase-separating prion-like domains:^36^ (S_9_Y)_15_, (S_4_Y)_30_, (SSY)_50_, (YS)_75_, (YYS)_50_, and Y_150_. Sequence features are shown graphically in Fig. 1b, and corresponding phase diagrams are shown in Fig. 1e. As expected, we predict systematically higher critical solution temperature as hydrophobicity increases. All simulated LCDs have a fraction of aromatic (“sticker”) residues *f*_h_ ≈ 0.14, and the range of critical temperatures observed of LCDs falls roughly within the range of *T*_c_ measured for the YS variants with *f*_h_ = 0.1 and *f*_h_ = 0.2.

To probe the effect of sequence patterning, we design three additional YS variants at a hydrophobic fraction *f*_h_ = 0.20, as the corresponding uniform sequence (S_4_Y)_30_ displayed the closest phase behavior to the LCDs with *f*_h_ = 0.14. We then alter the blockiness of the sequences: (S_12_Y_3_)_10_, (S_24_Y_6_)_5_, and S_120_Y_30_. In addition, we generate a randomly scrambled sequence, (S_120_Y_30_)^scr^, with the same composition. Graphical representations of these sequences are shown in Fig. 1c along with their measured blockiness *f*_B_ (see Methods), and corresponding phase diagrams are shown in Fig. 1f. The critical temperatures and phase boundaries of these patterning variants are all similar to those of LCDs except for those corresponding to (S_24_Y_6_)_5_ and S_120_Y_30_, the two blockiest sequences. These latter sequences form micelles instead of phase-separated condensates.

In addition to characterizing the phase behavior of the sequences, we investigate how molecular sequence affects condensate material properties by computing droplet surface tension. We measure the surface tension of each condensate *via* direct-coexistence simulations in the slab geometry^48^ at 0.9*T*_c_, where *T*_c_ is the critical solution temperature. We find that surface tension increases with LCD sequence length (Fig. 1g). Longer chains likely enhance surface tension both through confinement and entanglement effects, and by virtue of their larger conformational entropy in the dense phase, with the stability of the dense phase continually reinforced by the enthalpy of abundant transient intermolecular interactions.^49^ On the other hand, condensates formed by uniformly patterned YS sequences show that higher sequence hydrophobicity is proportional to surface tension (Fig. 1h), indicating that hydrophobicity plays a strong role in modulating material properties. The effect of hydrophobicity is likely obscured in simulated LCD condensates due to the dominant effect of sequence length with respect to minor variations in LCD hydrophobicity. Compared with a similar simulation study conducted in ref. 43, which found that a minimum hydrophobic content of 60% is required for phase separation of binary associative polymers with *n* = 20 beads, we find that sequences with *n* = 150 aa reliably phase separate with a hydrophobic content above 10%. This suggests that longer polymers can afford to have lower concentrations of associative sites while still forming stable, phase-separated droplets. The LCD sequences studied here have fewer than ≈5% charged residues (*cf.*Fig. 1a), so we do not expect sequence charge distributions to produce electrostatic effects contributing substantially to droplet stability or surface tension. The patterning variants shown in Fig. 1i demonstrate that surface tension decreases with increased sequence blockiness, proceeding in order from (S_4_Y)_30_ to (S_120_Y_30_)^scr^ and (S_12_Y_3_)_10_ (see Fig. 1c for sequence blockiness *f*_B_). However, the formation of micelle structures, shown in Fig. 1i, prevents us from properly calculating the surface tension for (S_24_Y_6_)_5_ and S_120_Y_30_. While the YS model enables a controlled study of sequence effects, the LCD trends suggest that the emergent material properties of the condensate are shaped by an interplay between sequence length and hydrophobicity. Together, these results point to nontrivial but interpretable relations between sequence composition, condensate phase behavior, and macroscopic material properties.

### 1.2. Small-world connectivity in LCD and YS condensates varies with length and is independent of sequence composition

To properly investigate the microstructure underlying condensates, we simulate 216 copies of each sequence in an isotropic box in the NVT ensemble at *T* = 0.9*T*_c_ and system density *ρ* = 0.05 g cm^−3^. The isotropic simulation cell permits the formation of spherical droplets at equilibrium, enabling graph-theoretic analyses on networks of finite size. In network representations of condensate microstructures, individual molecules are represented as single nodes, and interacting nodes are connected with unweighted and undirected edges (right panel in Fig. 2a). Specifically, we assign edges between two interacting molecules (*i.e.*, LCDs or YS sequences) A and B when the interaction potential energy falls below a threshold of −5*k*_B_*T*. The scalar multiple of *k*_B_*T* tunes the strength of the energetic criterion to unveil more transient (lower multiples) or more long-lived (higher multiples) networks representing the condensate microstructure. In the Mpipi model, the interaction strength encoding the strong hydrophobic Y–Y interaction is 0.42 kcal mol^−1^, which is slightly smaller than the thermal energy *k*_B_*T* = 0.59 kcal mol^−1^ at 300 K. A typical distribution of intermolecular interaction energies between pairs of A1-LCD molecules at 0.90*T*_c_ is shown in Fig. 2b, where 68% of all recorded intermolecular interactions are stronger than the thermal energy *k*_B_*T*, 33% of all interactions are stronger than 5*k*_B_*T*, and only 14% of all interactions are stronger than 10*k*_B_*T*. Considering that the 5*k*_B_*T* threshold represents an interaction energy greater than five instances of strong, hydrophobic Y–Y interactions, the results reported in the text and figures use 5*k*_B_*T* as a reasonable threshold to define relatively stable interactions and ensure that edges in computed networks represent long-lived associations within the microstructure.

Using this procedure, we find that condensates formed by LCDs and non-blocky YS sequences, including a hydrophobic homopolymer, consistently display small-world network microstructures that span the dense phase (Fig. 2d and e). Small-world networks are formally characterized by high clustering coefficients and low average shortest pathlengths.^50,51^ We quantify network small-worldness using the graph-theoretic estimators *σ*_sw_ and *ω*_sw_, which measure the average clustering coefficient (*C*) and average shortest pathlength (*L*) between arbitrary nodes in the graph.^52–55^ The equations used to compute these graph parameters are described in the Methods. Values of 0 < *σ*_sw_ < 1 indicate that clustering is low or average shortest pathlengths are long compared to equivalent Erdős–Rényi (ER) random graphs, and *σ*_sw_ ≈ 1 indicates that the network is organized like an ER random graph. Characteristic small-world values *σ*_sw_ > 1 come from high clustering coefficients and average shortest pathlengths that are shorter than or comparable to those in ER random graphs. The second estimator *ω*_sw_ is bounded between −1 and 1, where *ω*_sw_ = −1 corresponds to a regular, lattice-like graph structure and *ω*_sw_ = 1 corresponds to a random-graph structure. The small-world region *ω*_sw_ ≈ 0 describes a graph structure that is both highly clustered—like regular lattices—and has short average path lengths, like ER random graphs.^50,54^ As seen in Fig. 2d–f, we define a “small-world” region −0.3 ≤ *ω*_sw_ ≤ 0.3 as roughly the middle third of the range of *ω*_sw_ values, which ideally captures small-world networks (*ω*_sw_ ≈ 0) with a roughly 1-standard deviation margin of error. The balance of high clustering and short path lengths underlies the resilience and conduciveness of the small world network to efficient, high-fidelity transfer: most nodes are well-connected to local nodes in clustered “neighborhoods” (graph “cliques”), and these neighborhoods are globally linked through a small subset of highly connected “hub” nodes that act as highways mediating pairwise node relations through shortest paths.

Fig. 2d shows that all LCD condensates exhibit microstructures defined by small-world interaction networks with characteristic *σ*_sw_ > 1 and *ω*_sw_ ≈ 0 values. These results are consistent with those from lattice simulations,^10^ which found that single-component A1-LCD condensates have interaction networks exhibiting small-world topologies. LCD simulations reveal that network small-worldness and droplet surface tension are both proportional to sequence length (Fig. 1g and 2d, respectively). Strikingly, simulations of non-blocky YS sequences show that microstructure small-worldness is consistent across an order-of-magnitude change in hydrophobic content (Fig. 2e). While the surface tension of the non-blocky YS variants scales proportionally with sequence hydrophobicity (Fig. 1h), the graph-theoretic measures of small-world microstructure are not significantly different across the range of sequence compositions studied. In fact, the FUS-LCD and all non-blocky YS variants appear to have small-world parameters within error of each other, and these sequences are the closest in length (*n* = 163 aa for FUS-LCD and *n* = 150 aa for the YS variants). This suggests that small-worldness may be an intrinsic feature of discretely sticky polymers forming fluctuating, transient intermolecular associations, provided that the polymers are long enough and sticky enough to phase separate. This opens the design space available to the cell, allowing cells to stably condense polymers with varying interaction modes (*e.g.*, π–π, electrostatic, or hydrophobic attraction) and tune droplet material properties without grave concern for phase separation or interaction network stability.

The patterning variants shown in Fig. 2f suggest that increased sequence blockiness disrupts the small-world connectivity of the microstructure. Indeed, the blockiest sequences (S_24_Y_6_)_5_ and S_120_Y_30_ form micelles instead of phase separating. The effects of heterogeneous interactions are most dominant in these systems, and remarkably, these sequences show the greatest deviation from ideal small-worldness: their *ω*_sw_ values lie outside the range of small-world-like *ω*_sw_ ≈ 0 and indicate that these assemblies yield lattice-like interaction networks with high global clustering, likely due to packing in the hydrophobic micelle cores. While it is tempting to classify these networks as small worlds due to their *σ*_sw_ > 1 values, we note that an inherent weakness of both estimators *σ*_sw_ and *ω*_sw_ is their tendency to overrepresent the importance of graph clustering,^54^ which emerges naturally in our systems due to the spatial embedding of the network. Notably, the shortest LCD TIA1-LCD and the block-copolymer S_120_Y_30_ appear to have *ω*_sw_ values within error of each other (see Fig. 2d and f), though the reduced small-world character of TIA1-LCD is likely due to its short sequence length. As these quantities are widely accepted and used to describe small-worldness in the network literature, we continue to use them here to qualitatively compare the connective microstructures underlying a range of condensate compositions. Together, our results demonstrate how molecular sequence impacts phase separation and condensate material properties while preserving the small-world microstructure across a range of compositions.

### 1.3. Molecular hubs and cliques are spatially segregated

To further characterize condensate microstructure using underlying molecular networks, we analyze the spatial distribution of small-world topological features at the single-molecule level. Small-world networks rely on “hubs”, members of a small subset of highly connective nodes, to lower average pathlengths by mediating many of the shortest paths connecting arbitrary node pairs. Small-world networks also contain “cliques”, locally fully connected neighborhoods of nodes.^56^ In the context of biomolecular condensates, cliques are clusters of closely interacting macromolecules. Clique substructures tend to be bridged to other cliques through hubs, which promotes efficient flow and network resilience—crucial properties of small-world topologies that drive their adoption in engineered and natural settings.^56^

**Fig. 3:**
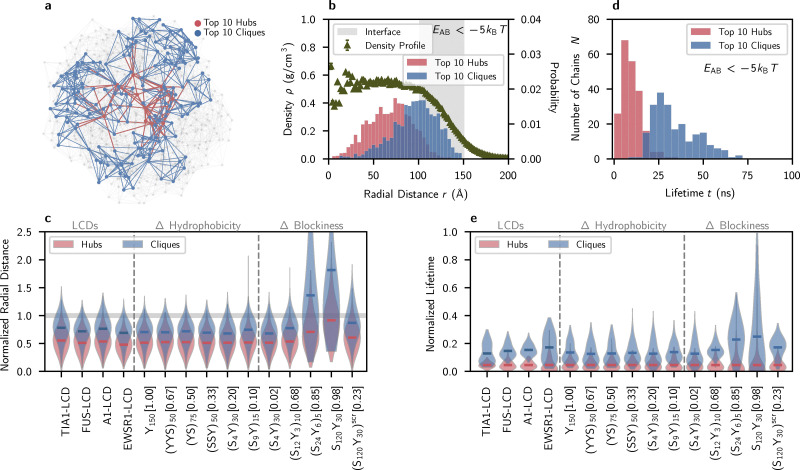
Network hubs and cliques are spatially and temporally distinct. Constructed interaction networks and graph-theoretic analyses use an energetic threshold of 5*k*_B_*T* to define network edges representing long-lived intermolecular associations. (a) Graph representation of an A1-LCD condensate (512 chains). Hubs are colored red and cliques are colored blue. Molecules colored gray are neither hubs nor members of the largest cliques. (b) Spatial distribution of hub molecules and clique molecules within a simulated FUS-LCD condensate (216 chains, *T* = 270 K). The clique molecules are closer to the interface than hub molecules. A radial distance of zero represents the center of mass of the condensate. The density profile is shown as triangles and the interface region is shaded in gray. An analogous profile from a simulation of 3375 FUS-LCD chains at *T* = 300 K is shown in Fig. S6; the observed distribution of hubs and cliques is persistent in larger systems and at higher temperatures. (c) The spatial distribution of hubs and cliques for all simulated sequences in terms of radial distance, normalized by the distance from each condensate's center of mass to its interface (overlaid grey bar). (d) The lifetime distribution of hub molecules and clique molecules are shown over 200 nanoseconds for FUS-LCD (216 chains, *T* = 270 K). Hub molecules are transient; the majority of hub molecules remain hubs on relatively short timescales, while molecules within cliques remain members of cliques for substantially longer periods of time. (e) Distributions representing the normalized lifetimes of hub molecules and clique molecules are shown for all simulated sequences.

We thus examine the spatial distributions of hub molecules and clique molecules in dense phases. The typical distributions of hub and clique molecules in the graph representation are depicted in Fig. 3a. Hubs are identified by high betweenness centralities *C*_B_, a measure of the extent to which a single node lies along shortest paths between arbitrary node pairs in the graph. Detailed calculations are shown in the Methods. Here, the top 10 hub molecules (*i.e.*, highest betweenness centrality *C*_B_) are colored in red and the 10 largest cliques are shown in blue. Next, we quantify the spatial distribution of hubs and cliques in the condensates. As shown for simulations of FUS-LCD in Fig. 3b, mass density profiles are roughly uniform within simulated condensates, featuring well-defined interfaces. However, despite homogeneous density profiles, the distributions of hub molecules and clique molecules are distinctly heterogeneous: cliques are located closer to the interface than are hubs, which are distributed throughout the volume of the condensate. We further confirm that such distributions are not a special case for FUS-LCD but are generic features of condensates formed by LCDs and uniformly patterned YS variant sequences, as characterized in Fig. 3d. We also confirm that this effect is not due to finite-size effects by simulating an analogous system composed of 3375 copies of FUS-LCD, shown in Fig. S6. We find that the observed distribution of hubs and cliques is persistent in larger systems.

Additionally, we find that the spatial distributions of hubs and cliques appear similarly conserved across a range of sequence patterns. Between (S_4_Y)_30_, (S_120_Y_30_)^scr^, and (S_12_Y_3_)_10_, greater sequence blockiness leads to slightly more pronounced distinctions between the spatial distributions of hubs and cliques (Fig. 3d), but the general spatial delineation persists. The blockiest sequences (S_24_Y_6_)_5_ and S_120_Y_30_ do not phase separate and instead form micelles with core–shell architecture (Fig. 2c). Hubs and cliques identified in micelles are not meaningful organizing features, as molecular packing in the hydrophobic micelle core enables global connectivity and elevated local clustering simultaneously. Due to this, no conclusive relation between sequence blockiness and hub–clique mesoscale inhomogeneity can be determined.

Collectively, our findings reveal that the small-world microstructure of non-blocky LCD-like condensates predictably encodes mesoscale heterogeneities within dense phases, consistently organizing molecules into distinct spatial regimes of interactivity over a range of sequence compositions and patterns. Molecules central to dense phases act as hubs, contributing to global network connectivity, while molecules near the interface tend to form clique clusters defined by tight local associations. This spatial partitioning of network roles reflects a non-random, emergent organization even in single-component condensates, offering a mechanistic link between network topology and spatial patterning. Notably, our results recapitulate previous experimental observations of nanoscale molecular clustering at the interfaces of multicomponent condensates,^40^ suggesting that the emergence of spatial inhomogeneities from the microstructure is broadly conserved in phase-separated macromolecular assemblies. These insights emphasize the importance of treating the condensate interface not as a passive boundary but as a functionally and structurally distinct region, whose qualities strongly influence condensate material properties, biological function, and aging behavior.

### 1.4. Molecular hubs and cliques exhibit distinct lifetimes

Given that networked microstructures lead to pronounced spatial inhomogeneities in condensates, an important open question is whether these microstructures also engender dynamical inhomogeneities, or differences in the temporal stability of network roles. Such inhomogeneities, especially as pertaining to interfacial properties, could have direct implications for condensate material properties. To explore this, we analyze the lifetimes of hubs and cliques within LCD and YS condensates to study the dynamics of the microstructure (see Methods). We measure molecular lifetimes based on their network identities: we quantify the fraction of time that a molecule is identified as a hub or as belonging to a clique over a continuous 200 ns trajectory sample. For FUS-LCD, we find that cliques exhibit significantly longer lifetimes than hubs (Fig. 3c). In fact, for all LCDs probed, individual molecules very scarcely serve as connective hubs for more than 1–2 ns in our simulations, while members of cliques remain in those cliques for substantially greater time fractions (Fig. 3e). This clear temporal separation of hubs and cliques is also observed for non-blocky YS sequences (Fig. 3e), and the behavior appears to be conserved across all uniformly patterned YS variants. Despite their longevity, molecular transfer in and out of clique clusters is still visible in Fig. 3e, namely *via* the lack of clique molecules that remain in their cliques for any greater than ≈40% of the trajectory sample.

YS pattern variants suggest that sequence blockiness may be weakly related to clique longevity: the blockiest phase-separating sequence (S_12_Y_3_)_10_ displays the clearest distributional distinction between hub and clique lifetimes, and hub and clique distributions overlap more as sequence blockiness decreases (Fig. 3e). Further, LCD condensates show that clique lifetimes are dependent on sequence length and diversity (Fig. 3e)—effects not captured in the binary YS sequences. For example, EWSR1, the longest and least hydrophobic LCD tested, exhibits both longer clique lifetimes and a broader distribution of clique lifetimes compared to other LCDs. TIA, the shortest, most hydrophobic, and least uniformly patterned LCD studied, has a broad and bimodal distribution of clique lifetimes. While the general temporal distinction between hubs and cliques in LCD and YS condensates remains conserved, these results imply that the properties of molecular clusters at interfaces, including their stability and local dynamics, may be fine-tuned with sequence patterning.

Taken together, these results suggest that the condensate microstructure obeys counterintuitive dynamics. The backbone of the small-world network consists of highly associative hub molecules who rapidly interchange roles, while the peripheral networks of local interactions between clique molecules are macroscopically more time-stable. This “decentralization” of hublike connectivity in the microstructure may represent a physical mechanism of resilience against network failure due to aberrant single-chain behavior. Moreover, experiments have shown that nanoscale molecular clustering is linked to reduced molecular diffusion in dense phases.^40^ The relative stability of clique clusters, along with their localization to condensate interfaces, suggests that clustering may play a role in selective molecular recruitment and dynamic confinement. These results provide a framework for understanding and interpreting condensate function and mesoscale inhomogeneity as emerging from interaction networks, which importantly remain robust to large variations in sequence composition and patterning.

### 1.5. Conformations of molecules in biomolecular condensates are dependent on network topology

**Fig. 4:**
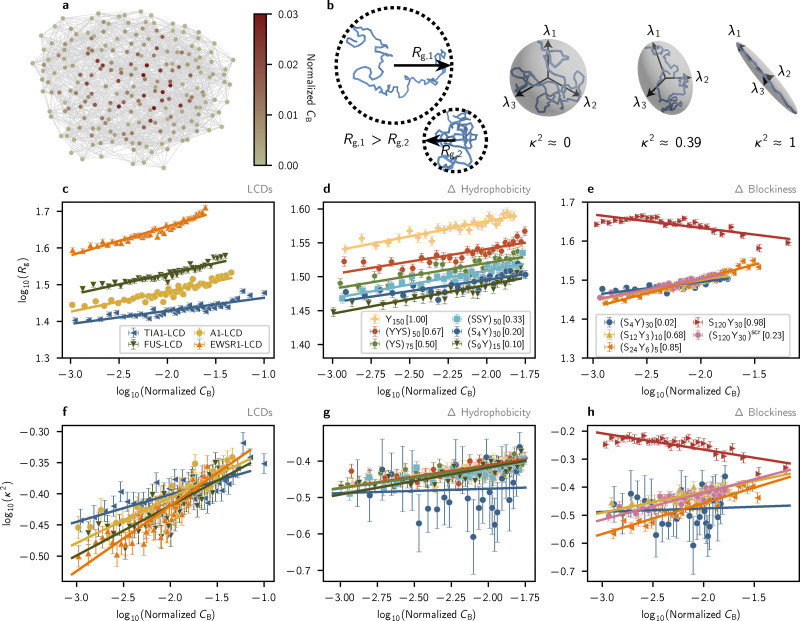
Single-chain radius of gyration *R*_g_ and shape anisotropy *κ*^2^ follow power-law relationships with molecular connectivity in interaction networks. All graph-theoretic analyses are performed by constructing interaction networks using the energy threshold 5*k*_B_*T* to define edges representing long-lived intermolecular associations. (a) A graph of an A1-LCD condensate. Nodes are colored based on their betweenness centrality *C*_B_. (b) (left) Schematic representations of chain radius of gyration *R*_g_. (right) Schematic representations of the eigenvectors *λ* of the gyration tensor and values of the scale-invariant shape anisotropy parameter *κ*^2^ for distinct chain conformations. *κ*^2^ = 0 represents a perfect, radially isotropic sphere, *κ*^2^ ≈ 0.39 corresponds to an ideal-chain conformation, and *κ*^2^ = 1 describes a perfectly anisotropic elongated chain. (c)–(e) Possible power-law relations between the betweenness centrality *C*_B_ and radius of gyration *R*_g_, indicated by linear fits in log_10_–log_10_ space. (f)–(h) Possible power-law relations between the betweenness centrality *C*_B_ and relative shape anisotropy *κ*^2^.

Recent work has highlighted that LCDs adopt heterogeneous conformational ensembles within condensates, with molecular properties that vary depending on the local microenvironment. In particular, differences in average molecular size (*i.e.*, radius of gyration *R*_g_) have been reported between proteins at the interface and those in the condensate core.^10,19,21^ In parallel, graph-theoretic analyses have been applied to reveal inhomogeneities in networks of molecular interactions underlying LCD condensates.^10–12,18,41^ Building on these observations, we investigate whether molecule-scale conformational properties are systematically related to network features (Fig. 4a) within the microstructures of LCD-like condensates. Specifically, we measure the radius of gyration *R*_g_ and shape anisotropy *κ*^2^ of proteins in the condensates. *R*_g_ provides insight into the average molecular size, while *κ*^2^ effectively describes the deviation of polymer shape from a perfect sphere^57–59^ (Fig. 4b).

We first compare single-molecule *R*_g_ against molecular betweenness centrality *C*_B_ (normalized; see Methods). Recall that *C*_B_ quantifies the importance of a molecule (*i.e.*, node) based on how often it lies on the shortest paths between other molecules, such that a higher *C*_B_ indicates a more central, globally influential position within the network. We find that *R*_g_*versus C*_B_ in log_10_–log_10_ space yields a positive linear relationship (Fig. 4c) for LCDs, suggesting a consistent power-law relationship:

1 *R*_g_ = *aC*^*k*^_B_ → log_10_(*R*_g_) = *b* + *m* log_10_(*C*_B_).

This power-law-like behavior is also observed in condensates formed by uniformly patterned binary YS sequences (Fig. 4d). In both cases, individual macromolecules become more expanded as their network centrality increases, indicating that condensate microstructures reliably encode inhomogeneities at the single-molecule scale. Among LCD sequences, Fig. 4c shows that longer chains such as EWSR1 exhibit larger radii of gyration, consistent with expected scaling effects. For uniformly patterned YS sequences of fixed length, *R*_g_ increases monotonically with *C*_B_ with similar linear slopes across all variants (Fig. 4d). Comparing between these sequences reveals that the baseline expansion (intercept) increases with sequence hydrophobicity, resulting in a clear ordering of YS variants by both *R*_g_ and hydrophobic fraction *f*_h_. While higher hydrophobicity is often associated with chain compaction, the crowded dense phase environment likely leads to entropic favorability of intermolecular contacts over intramolecular contacts.^49^ In a densely packed “solvent” composed of fluctuating copies of a single molecule, greater sequence hydrophobicity enthalpically promotes intermolecular interaction. The translational entropy cost of an intermolecular interaction is lower than the conformational entropy cost of an intramolecular interaction, resulting in a preference for expanded configurations at higher hydrophobicity. This phenomenon has previously been reported for simulations of RNA condensates^60^ and in lattice simulations of flexible polymers with heterotypic binding motifs,^49^ underscoring its relevance to our findings. Together, these results suggest that the phase separation of discretely sticky polymers is stabilized by enthalpy gained from abundant intermolecular interactions and by the lower entropic cost of intermolecular contacts, compared to intramolecular contacts, in the dense phase. We demonstrate that the entropic mechanism underlying chain expansion in the dense phase persists over an order-of-magnitude change in sequence composition and droplet surface tension, with no perturbations to the condensate microstructure.

*R*_g_ profiles for YS sequences with fixed composition and varying patterning appear to corroborate this result (Fig. 4e), with all but the blockiest sequence (S_120_Y_30_) having *R*_g_ values collapsing onto the same curve. However, the most blocky sequence S_120_Y_30_ exhibits an almost inverse trend, where increased centrality *C*_B_ is negatively correlated with molecular radii of gyration. This result may be attributed to the packing of hydrophobic poly-Y tails in the hydrophobic core of the micelle formed by S_120_Y_30_.

Similar to *R*_g_, the molecular relative shape anisotropy *κ*^2^ also appears to follow power-law relationships with molecular *C*_B_ for LCDs and non-blocky binary YS sequences, albeit with smaller coefficients of determination *R*^2^ (Fig. 4f and g). *κ*^2^ is a scale-invariant quantity, ranging from *κ*^2^ = 0 at the limit where polymers adopt a spherically isotropic conformation to *κ*^2^ = 1 at the limit where they are completely linear (Fig. 4b, right panel). LCD simulations reveal that greater slopes in the *κ*^2^(*C*_B_) relationship correlate with greater small-worldness in the network and greater surface tension (Fig. 1g, 2d and 4f), though this trend is not obvious for the YS sequences and likely arises from sequence complexity not captured in the YS variants (Fig. 4g and h). Previous simulation studies have reported the average relative shape anisotropy of individual polyampholytes in condensed phases,^19^ with dense-phase *κ*^2^ consistently between 0.42 and 0.44 invariant to changes in sequence. However, our results show that LCDs in dense phases exhibit a range of conformations that map onto their connective roles in the condensate microstructure. Compared to the ideal-chain *κ*^2^ = 0.39, molecules with low *C*_B_ adopt slightly collapsed conformations (*κ*^2^ ≈ 0.28) while highly connected hublike molecules become slightly expanded (*κ*^2^ ≈ 0.48).

In summary, we demonstrate the potential to interpret complex single-molecule inhomogeneities in dense phases using principled analyses of condensate microstructures. We predict the presence of continuous quantitative relationships bridging single-molecule conformation and network centrality, whose parameters depend on sequence composition and length. Notably, we find that hublike character corresponds to maximal expansion among the conformations assumed by macromolecules in all condensates formed by LCD and non-blocky YS sequences. Simulations of YS variants show that greater sequence hydrophobicities promote chain expansion and increase droplet surface tension while preserving the small-world-like qualities of the microstructure. Collectively, our results demonstrate that LCD-like sequence properties influence both microscopic and macroscopic condensate features while leaving the dense-phase microstructure intact. Remarkably, molecular connectivity within the conserved small-world microstructure reliably encodes conformational and mesoscale inhomogeneities of the dense phase across all phase-separating sequences studied.

### 1.6. Dynamics of molecules in biomolecular condensates are dependent on network topology

In addition to linking molecular conformation and condensate microstructure, we characterize the dynamics of individual molecules inside condensates. In particular, we investigate scaling relationships between the displacement of molecular centers of mass (|Δ**r**|) and betweenness centrality (*C*_B_). The computation and normalization of these quantities are described in the Methods.

**Fig. 5:**
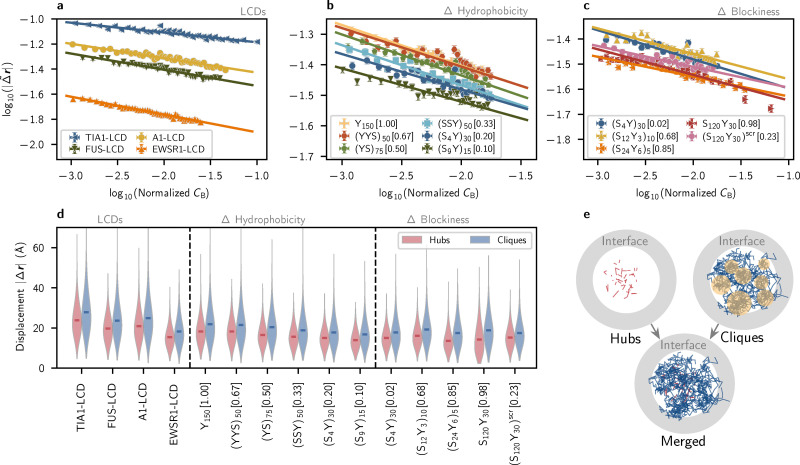
Dynamics of molecules within single-component condensates is correlated with network topological organization. (a)–(c) Normalized instantaneous displacements |Δ̃**r**| of single chains in condensed phases exhibit strong negative correlations with node betweenness centrality *C*_B_ within molecular graphs. (d) Displacements within consistent time intervals are compared for hub molecules and clique molecules. (e) A 2-dimensional visualization of the trajectories of hub molecules (red) and clique molecules (dark blue) in a FUS-LCD condensate, plotted as lines when their hub or clique-member status is contiguous in time. The phase interface is shown as a gray circular band. Locally confined regions of clique molecules are indicated by yellow circles.

Similar to the conformational properties *R*_g_ and *κ*^2^, we find that normalized molecular displacement |Δ̃**r**| follows an apparent power-law relationship with *C*_B_, exhibiting linear correlations in log_10_–log_10_ space (Fig. 5a–c). Given that the LCD sequences have similar hydrophobicities, the LCD data mainly show that molecular size strongly impacts displacement; the shortest sequence TIA1 exhibits the fastest dynamics and the longest sequence EWSR1 exhibits the slowest dynamics (Fig. 5a). YS sequences show that increased sequence hydrophobicity is associated with greater overall molecular displacement when compared at 0.9*T*_c_ (Fig. 5b). This trend suggests that, at a fixed relative distance from the critical temperature, more hydrophobic sequences experience a more favorable environment (*i.e.*, better solvent quality) within the dense phase, promoting increased mobility and shaping the internal dynamics of the condensate. These results reveal that condensate microstructure is linked to single-molecule dynamics as well as conformational properties, supporting its explanatory power for both static and dynamic condensate phenomena.

To assess whether the observed dynamic and conformational features arise from polymer crowding in addition to sequence-specific interactions and networked microstructures, we compute the dimensionless density *ϕ*_rel_^61,62^ of each LCD and YS condensate (see Methods). We find that LCDs and non-blocky YS sequences all yield effective packing densities in the range 1 < *ϕ*_rel_ < 1.2 (Fig. S19a and b), indicating that these condensates lie just beyond the overlap threshold, where crowding and entanglement may begin to influence molecular dynamics and microstructure. However, no particular ordering is observed for the packing density *ϕ*_rel_ with respect to sequence hydrophobicity *f*_h_ or blockiness *f*_B_ (Fig. S19b and c), unlike the clear ordering of trends in molecular conformations (Fig. 4c and d), single-molecule dynamics (Fig. 5a and b), and condensate surface tension (Fig. 1g and h) observed with respect to sequence features like *f*_h_. These results suggest that polymer crowding alone does not account for the observed phenomena, and that sequence length and hydrophobicity remain the primary drivers of condensate microstructure and material properties.

Experimental and simulation studies have characterized the diffusion of LCDs within condensates.^63,64^ More recent experimental studies have shown that the dense phases of FUS–RNA condensates contain “nanodomains”, densely interacting molecular clusters that decrease local diffusivity without a secondary phase separation.^40^ To study this effect and its relation to the small-world microstructure, we additionally analyze the local movements of hub and clique molecules in our systems. On short timescales, the displacements of clique molecules are consistently greater than those of hubs in both LCD and YS-variant simulations (Fig. 5d). This can be intuitively explained with the observed degree distribution of hub molecules and clique molecules within interaction networks: hub molecules associate with a greater number of partners, thus confining their motion to a greater extent (Fig. S11–S14). When combined with our previous analysis, which reveals that cliques have longer lifetimes (Fig. 3d and e), we conclude that the “faster” motion of clique molecules is best described as a form of local vibration. Indeed, when we trace the displacement of molecules in cliques and hubs, the motion of clique molecules is highly localized (see the 2D projections of molecular motion in Fig. 5f). Thus, while cliques experience relatively larger displacements than hubs, these displacements remain confined to microscale regions near the interface. Such confinement of clique motion is reminiscent of the nanodomains described in ref. 40, as well as of recent experiments reporting highly interactive hydrophobic “hotspots” in A1-LCD condensates.^12^ These results support the hypothesis that nanoscale clusters at interfaces might contribute to selective molecular recruitment and confinement through local modulation of material properties.

## 2. Discussion

Macroscopically, biomolecular condensates often appear as homogeneous structures in both experimental and simulated reconstitution. However, evidence suggests that even single-component condensates can exhibit inhomogeneities in their microstructure.^10–12,15,37^ The molecular networks underlying condensates have also been theorized to play crucial roles in shaping their material properties and functions. Understanding how the material properties of biomolecular condensates arise from complex microstructures and molecular features is thus an emerging area in the field.

In this work, we leverage residue-resolution molecular dynamics simulations alongside graph theory to reveal that LCD-like sequences inherently form stable, small-world microstructures despite large, sequence-encoded variations in molecular conformation and dynamics as well as macroscopic material properties. We systematically characterize the microstructures of condensates formed by the low-complexity domains (LCDs) of key phase-separating proteins (hnRNPA1, FUS, EWSR1, and TIA1) using network-based approaches. To assess the generality of our findings, we also characterize condensate systems composed of binary sequences with varying fractions of hydrophobic Y residues and polar S residues. These YS variant systems represent associative heteropolymers with varying propensities for forming percolated networks within condensates.

In agreement with previous lattice-based simulations of A1-LCD,^10^ we find that condensates formed by biological LCDs and generic non-blocky YS sequences, including a hydrophobic homopolymer, consistently adopt microstructures well described as small-world networks. We discover that condensate microstructure and droplet surface tension are consequences of sequence length and hydrophobicity, and remarkably, we show that the graph-theoretic properties of the network microstructure are consistent over an order-of-magnitude change in sequence composition using the YS sequences of fixed length. While the microscopic and macroscopic characteristics of the droplet, including molecular conformations, dynamics, and surface tension, vary predictably with sequence composition and patterning, the small-world microstructure and the clustered organization of the dense phase persists.

Small-world networks contain two major topological features, “hubs” and “cliques”. Cliques are densely connected groups of nodes (here, molecules) representing fully connected subgraphs within the network. The cliques themselves are efficiently bridged through hub nodes, which are highly connective and reduce average shortest path lengths between arbitrary node pairs in the network. We consistently find that hubs are positioned closer to the condensate center, while the largest cliques are located near the interface. Such spatial organization of network features enables efficient transmission of internal stresses to clique clusters at the interface, potentially influencing the surface tension underlying droplet stability. Condensate microstructures, then, may be viewed as spatially embedded networks whose complex properties stably enable a rich array of sequence-encoded material behaviors.

Our work also elucidates how mesoscale inhomogeneities arise from internal network architectures. Hubs and cliques represent two distinct regimes of molecular interactivity within the microstructure: hub molecules are responsible for high global connectivity and are marked by expanded conformations, while clique clusters are nanoscale regions of elevated local associativity marked by interfacial localization and confined molecular motion. In addition to the spatial distinction observed between hubs and cliques, we find that hub molecules and clique molecules have distinct lifetimes: the molecular identities of connective hubs change rapidly, while members of cliques tend to remain in those cliques over longer timescales due to the formation of stable, fully-connected subnetworks. These results are highly counterintuitive. Network hubs are critical for network stability and often form many strong intermolecular associations, but their role is shown to be transient. Network cliques represent molecular clusters at the interface marked by maximal local connectivity, making them sensitive to minor perturbations, yet clique structures are demonstrably more time-stable. Such inverted behavior may be a crucial feature of resilience and function in LCD condensates: transient hubs allow decentralization of the backbone holding the network together, while stable clusters may modulate selective molecular recruitment and dynamic arrest by controlling the material characteristics of the interface.

At the single-molecule scale, we reconcile reports of conformational heterogeneity and connective heterogeneity in condensates by showing that the conformational properties of macromolecules are highly correlated with their connectivity within network microstructures. Interestingly, the radius of gyration (*i.e.*, average size) and shape anisotropy (*i.e.*, ranging from spherical to linear) of individual molecules are found to follow power-law-like relations to molecular betweenness centrality (*i.e.*, connectivity) within interaction networks. This behavior is conserved across a range of sequence lengths, compositions, and patterns. We find that increasing sequence hydrophobicity does not alter the nature or the slope of the relationship. Instead, similar to previous reports, increased hydrophobicity leads to greater overall chain expansion through an entropic mechanism, which corresponds to increased droplet surface tension independent of the organization of the microstructure. Further, we find that sequence hydrophobicity does not appear to alter conformational anisotropy in dense phases but sequence length strongly impacts the degree of chain extension and linearization with increasing connectivity. Lastly, we note that the blockiest sequences (S_24_Y_6_)_5_ and S_120_Y_30_ form micelles instead of phase separating. Curiously, the block-copolymer sequence S_120_Y_30_ exhibits relationships that are inverted relative to all other sequences, and we attribute this behavior to packing and disorder in the hydrophobic micelle core and periphery, respectively (Fig. S19c). This behavior is not representative of phase-separated macromolecules in solution.

It is striking that molecule-scale physical quantities are strongly correlated with betweenness centrality and not with node degree, the intuitive measure of polymer associativity. Betweenness centrality and degree are naturally weakly correlated (Fig. S7–S10), as a node with a greater number of associations has an increased likelihood of lying along shortest paths, *i.e.*, being globally connective. Indeed, on average, node centrality and degree vary similarly with increasing radial distance from the condensate center (Fig. S15–S18). However, high betweenness centralities do not require high degrees and *vice versa*. We find that the diverse conformational and dynamic characteristics assumed by single molecules in dense phases are most coherently viewed in a relationship with *C*_B_ and not with degree or radial distance. We take these findings to mean that shortest-path centrality is a critical organizing principle of the condensate microstructure, and further, that inhomogeneities at the single-molecule scale are more strongly related to emergent properties of internal networking than directly to the number of intermolecular bonds or to spatial location within the dense phase. The observed ubiquity of the small-world topology also suggests that the design space available to the cell is vast:^65^ LCD-like sequences with varying inter-residue attraction modes can all be co-condensed, and their sequences can be tuned to produce certain material or dynamical characteristics without concern for phase separation propensity and droplet stability. Taken together, these results corroborate the notion that small-world microstructures contribute to both microscopic properties—through the relations uncovered here—and macroscopic material properties, by enabling efficient internal stress transmission.

In addition to structural properties of macromolecules, we explore their dynamics in the context of the microstructure. We find that molecules become less dynamic as their betweenness centrality increases. This result is intuitive, as more expanded molecules with larger betweenness centralities are subject to greater confinement through dense intermolecular interactions in the condensate environment. Interestingly, this relationship also shows power-law behavior. As expected, we find that smaller molecules exhibit faster motions in the dense phase. Increasing sequence hydrophobicity also leads to greater overall displacements across the range of the relationship, further illuminating how sequence features tune the relation between condensate microstructure and molecular properties. We also analyze the effective packing density of each condensate to confirm that the observed structural and dynamic features of individual molecules arise primarily from sequence-encoded interactions and not from molecular crowding in dense phases, though a detailed study of the relation between sequence features and molecular packing may be warranted.

To our surprise, molecules in interfacial cliques exhibit slightly faster motions than hub molecules. Explicitly tracing their trajectories, however, reveals that clique molecules are spatially confined. This suggests that their movement is primarily characterized by local vibrations, whereas hub molecules exhibit motion across larger regions. Recent work on multicomponent condensates has identified nanodomains within condensates marked by elevated local connectivity, interfacial localization, and reduced molecular diffusion,^40^ all of which appear to be consistent with the cliques we observe in LCD and YS condensates. Other recent experiments have also reported highly interactive hydrophobic nanoclusters in A1-LCD condensates;^12^ although ref. 12 refers to these regions as “hubs,” their nanoclusters align with the “cliques” found in our systems according to graph-theoretic principles. Alongside these studies, experiments report that pathological liquid-to-gel transitions in condensates originate at their interfaces.^31,66^ Additionally, the formation of interfacial aggregates resembling amyloid fibrils were observed in the early stages of FUS condensate aging.^32,66^ The arresting effects of interfacial aggregation can, in turn, arrest the dynamics of the entire condensate by propagating through the small-world network structure. Thus, the stability of locally constrained, highly interactive molecular clusters near condensate interfaces may be linked to both function and dysfunction, enabling selective molecular recruitment and pathological aggregation.

A key limitation of this work is that the probed condensates are composed solely of disordered protein sequences that engage in transient interactions. In contrast, cellular condensates often include both disordered and structured components that dictate their form and function.^67^ The latter can mediate long-lived, high-affinity interactions that shape the underlying interaction networks, as recent studies suggest.^68,69^ Exploring systems with specific binding interactions—such as those involving folded protein domains or RNA—would therefore be an important next step. Nonetheless, our findings show that even simple systems of disordered protein regions exhibit striking microstructural inhomogeneities that give rise to complex biophysical behaviors. We further note that the small-world measurements employed here, namely *σ*_sw_ and *ω*_sw_, are to be treated as estimators. These measurements are designed to reflect both the spatial structure (*via* high clustering) and efficient communication (*via* low average pathlengths) of small-world networks by generating ensembles of equivalent random-like or lattice-like graphs for comparison. While these measurements are commonly accepted and used in the network literature,^52–54,56^ they are not robust to measurement errors or effects such as elevated clustering arising from the spatial embedding of our networks. A more rigorous characterization of network structure with stricter boundaries defining “small-worldness” would be useful to quantitatively verify our claims, though to our knowledge, no such characterization exists for small-world networks beyond the methods employed here.

Collectively, our results elucidate the rules by which molecular features encode rich microscopic and material properties of condensates while maintaining a conserved, networked microstructure. We demonstrate that LCD-like condensates consistently adopt small-world network microstructures that persist with large variations in sequence composition and patterning, molecular characteristics, and macroscopic properties such as surface tension. Further, we analyze the spatial and temporal characteristics of distinct network features, hubs and cliques, to interpret experimentally observed mesoscale inhomogeneities in the context of the microstructure. We finally uncover quantitative relationships that govern the distribution of molecular conformations and dynamics in dense phases from molecular network connectivity. These findings reveal complex, multiscale structure–property relationships in LCD condensates that provide general principles for designing soft materials with stable internal architectures and diverse material characteristics. We anticipate that our results can be extended to other biomolecular systems with varying residue–residue interaction modes to inform the design of synthetic condensates with programmable, interpretable composition and properties.

## 3. Methods

In this work, we study the single-chain characteristics, interaction network topologies, and surface tensions of single-component condensates formed by prion-like low-complexity domains (LCDs) using Mpipi—a residue-level coarse-grained model for disordered proteins.^42^ We further study the effect of sequence on multiscale properties by simulating single-component condensates comprising binary sequences of tyrosine (Y) and serine (S) residues. In the binary sequence simulations, we systematically vary sequence hydrophobicity (fraction of Y, *f*_h_) and blockiness (fraction of consecutive residues, *f*_B_) to investigate their impact on the topology of emergent interaction networks, as well as their downstream impact on surface tension as well as molecular conformation and dynamics.

### 3.1. LCD and binary sequences

We use the Mpipi model^42^ to simulate four biological phase-separating protein sequences: the low-complexity domain of the heterogeneous nuclear ribonucleoprotein hnRNPA1 (A1-LCD), the low-complexity domain of the fused in sarcoma protein (FUS-LCD), the low-complexity domain of the RNA-binding protein EWS (EWSR1-LCD), and the low-complexity domain of the T-cell intracellular antigen 1 (TIA1-LCD). The sequences are shown below.

**Table:** 

A1-LCD	GSMAS	ASSSQ	RGRSG	SGNFG	GGRGG	GFGGN
	DNFGR	GGNFS	GRGGF	GGSRG	GGGYG	GSGDG
	YNGFG	NDGSN	FGGGG	SYNDF	GNYNN	QSSNF
	GPMKG	GNFGG	RSSGG	SGGGG	QYFAK	PRNQG
	GYGGS	SSSSS	YGSGR	RF		
EWSR1-LCD	MASTD	YSTYS	QAAAQ	QGYSA	YTAQP	TQGYA
	QTTQA	YGQQS	YGTYG	QPTDV	SYTQA	QTTAT
	YGQTA	YATSY	GQPPT	GYTTP	TAPQA	YSQPV
	QGYGT	GAYDT	TTATV	TTTQA	SYAAQ	SAYGT
	QPAYP	AYGQQ	PAATA	PTRPQ	DGNKP	TETSQ
	PQSST	GGYNQ	PSLGY	GQSNY	SYPQV	PGSYP
	MQPVT	APPSY	PPTSY	SSTQP	TSYDQ	SSYSQ
	QNTYG	QPSSY	GQQSS	YGQQS	SYGQQ	PPTSY
	PPQTG	SYSQA	PSQYS	QQSSS	YGQQS	SFRQD
	HPSSM	GVYGQ				
TIA1-LCD	MINPV	QQQNQ	IGYPQ	PYGQW	GQWYG	NAQQI
	GQYMP	NGWQV	PAYGM	YGQAW	NQQGF	NQTQS
	SAPWM	GPNYG	VQPPQ	GQNGS	MLPNQ	PSGYR
	VAGYE	TN				
FUS-LCD	MASND	YTQQA	TQSYG	AYPTQ	PGQGY	SQQSS
	QPYGQ	QSYSG	YSQST	DTSGY	GQSSY	SSYGQ
	SQNTG	YGTQS	TPQGY	GSTGG	YGSSQ	SSQSS
	YGQQS	SYPGY	GQQPA	PSSTS	GSYGS	SSQSS
	SYGQP	QSGSY	SQQPS	YGGQQ	QSYGQ	QQSYN
	PPQGY	GQQNQ	YNS			

These LCDs are marked by a sequence distribution overrepresented in glutamine (Q), serine (S), glycine (G), and tyrosine (Y) residues. The polar uncharged residues Q, S, and G act as weakly interactive “spacers” along sequences, serving to segregate highly attractive “sticker” residues (particularly Y) uniformly along the sequence. TIA1-LCD also incorporates tryptophan (W) residues along the sequence that can enable “sticky” interactions with itself and tyrosine (Y) through π–π stacking of aromatic rings.

As for the binary YS sequences, we simulate chains with a constant length *n* = 150 to be close to the length of the LCD sequences described above. Furthermore, 11 sequence variants with different hydrophobic fractions are simulated and analyzed: (S_4_Y)_30_, (S_120_Y_30_)^scr^, (YS)_75_, Y_150_, (S_12_Y_3_)_10_, (S_24_Y_6_)_5_, (YYS)_50_, (S_9_Y)_15_, S_120_Y_30_, (SSY)_50_, and S_150_. Analogous to LCD architectures, each constructed variant distributes hydrophobic Y beads as evenly as possible along the sequence. These sequences represent a range of sequence compositions spanning an order-of-magnitude change in hydrophobicity. Among the sequences, S_150_ has the lowest hydrophobicity *f*_h_ = 0.00 and does not phase separate at *T* > 104 K; we have thus excluded this sequence from the results. Y_150_ has the largest hydrophobicity *f*_h_ = 1.00, and correspondingly, the highest critical temperature *T*_c_ = 945 K. In between, we cover a wide range of hydrophobicities: *f*_h_ = 0.10 for (S_9_Y)_15_; *f*_h_ = 0.20 for (S_4_Y)_30_, (S_4_Y)^scr^_30_, (S_12_Y_3_)_10_, (S_24_Y_6_)_5_, and S_120_Y_30_; *f*_h_ ≈ 0.33 for (SSY)_50_; *f*_h_ ≈ 0.67 for (YYS)_50_. Since *f*_h_ = 0.20 is similar to the hydrophobicity of LCD sequences (*f*_h_ ≈ 0.14), we vary sequence blockiness at this fixed composition, and we also scramble a sequence to further examine the effects of sequence patterning. This scrambled sequence is denoted (S_120_Y_30_)^scr^.

**Table:** 

(S_120_Y_30_)^scr^	SSSSS	SSSSS	SSYSS	YSYYY	YSSSS	SSSSS
	YSSSS	SSSSY	SSSSY	SSSSS	SSSYS	SSSSS
	SSSSS	SYSSY	YYSSS	SSSYS	YSYSS	SSSSS
	YSSSY	SSYSS	SSSSS	YSSSY	YSSSS	SYYSY
	SYSYS	SSSSS	SSSYS	SSSSS	SSSSS	YSSSS

The blockiness *f*_B_ is quantified by calculating the ratio of the number of actual Y–S and S–Y bonds *B*_act_ over the possible maximum number of Y–S and S–Y bonds *B*_max_,2
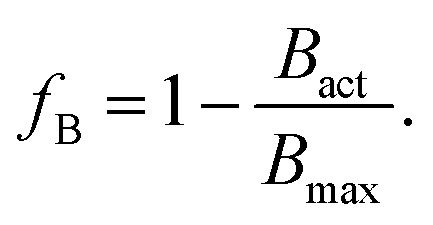
When *f*_B_ → 1, it means that there are fewer Y–S and S–Y bonds, indicating the increased blockiness. On the opposite side, the least blocky sequence maximizes the number of Y–S and S–Y bonds, thus *f*_B_ → 0.

### 3.2. Mpipi model

In Mpipi, each protein residue is represented by a single interaction site/bead. Each bead has an assigned mass, charge, molecular diameter, and other interaction parameters. The potential energy in the Mpipi model is taken as the sum of bonded and non-bonded interaction terms:

3 *E*_Mpipi_ = *E*_bond_ + *E*_elec_ + *E*_pair_.

Specifically, beads are bonded *via* harmonic springs:4
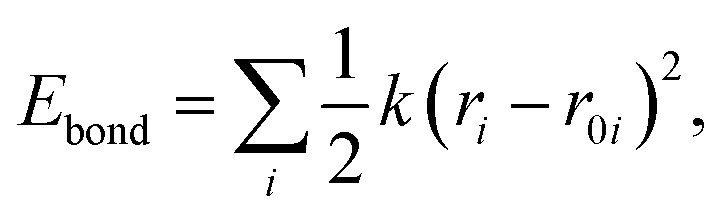
where the bond spring constant *k* is 19.2 kcal mol^−1^ Å^2^ and the equilibrium bond length is 3.81 Å.

Non-bonded interactions encompass long-ranged electrostatics, which are captured *via* a Coulomb term with Debye–Hückel screening,5
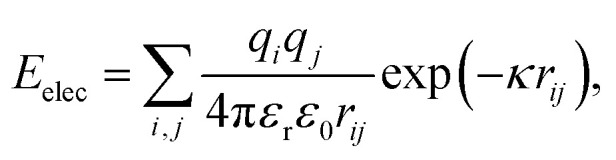
where *ε*_r_ = 80 is the relative dielectric constant of water and *ε*_0_ is the electric constant. The Debye screening length is set to *κ*^−1^ = 7.95 Å with a Coulomb cutoff of 35 Å.^42^ Non-bonded interactions also include short-ranged pairwise contacts, which are modeled *via* the Wang–Frenkel potential,^70^6
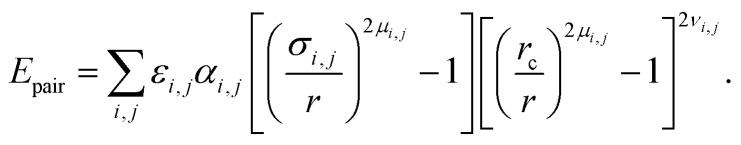


All model parameters are discussed in detail in ref. 38 and 42 and are provided in our GitHub repository (see Data availability). In Mpipi, the solvent is modeled implicitly; the model was parameterized by combining bioinformatics data and atomistic potentials-of-mean force calculations. Previous work has demonstrated that Mpipi accurately captures both single-chain properties and collective phase behaviors of disordered proteins.^38,42,71^

### 3.3. Molecular dynamics simulations

Implicit-solvent simulations are conducted in the NVT ensemble using the LAMMPS package.^72^ Each simulation consists of a single-component system comprising 216 copies of the corresponding LCD or YS sequence. While preserving the number of polymers across simulations leads to dense phases of variable sizes for polymers with differing lengths (*e.g.* TIA1-LCD *versus* EWSR1-LCD), we fix the number of polymers to enable meaningful comparison of graph structures and statistics.

First, NPT simulations are performed to accelerate the condensate formation process during the initial steps. A Berendsen barostat is used to apply an isotropic external pressure to the particles in each simulation cell, effectively overcoming the nucleation barrier and compressing the polymer chains into a condensed state. LCD simulations are compressed with a fixed isotropic pressure set to 100 atmospheres for 120 000 timesteps (d*t* = 10 fs) with a pressure damping parameter of 10 000d*t*. YS sequence variants are subject to a time-varying pressure that increased from 50 atmospheres to 100 atmospheres over a period of 30 000 timesteps (d*t* = 10 fs) with a pressure damping parameter of 100 000d*t*.

The simulation cells are then relaxed to a volume corresponding to a constant mass density *ρ* = 0.05 g cm^−3^ while preserving the condensed polymers in the center of the simulation cell. Production runs are then performed in the NVT ensemble at 0.9*T*_c_ and 0.95*T*_c_. The integration timestep is set to d*t* = 10 fs, and systems are simulated for 1 μs after condensate formation for equilibrium sampling. 1000 frames are sampled uniformly along equilibrium trajectories for each sequence. For each sampled frame in both simulation types, dense-phase centers of mass and single-molecule conformations are obtained using OVITO.^73^

### 3.4. Construction of interaction graphs

Interaction matrices representative of single static frames are constructed from particle position data, and we use an energetic criterion to ensure that the interaction energy of two chains a and b exceeds the thermal energy, *i.e.*, *E*_pair,ab_ < −5*k*_B_*T*, when recording an interaction. For *N* condensed polymers, a 2-dimensional interaction matrix (adjacency matrix) *M* = *N* × *N* is constructed, where *M*_ab_ is assigned as follows:7
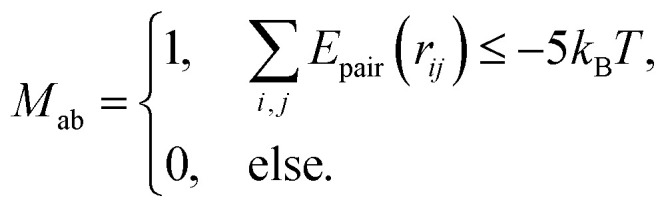
Here *i* and *j* index over each bead (residue/monomer) along respective protein chains a and b, and *r*_*ij*_ is the distance between monomers a and b. Finally, graph structures are generated with the NetworkX python package^74^ using binary interaction matrices as adjacency matrices *M*. Each node in a frame's representative graph represents a single protein chain, and unweighted, undirected edges are drawn between nodes if an attractive interaction between them is observed in that frame according to the above criteria.

Interaction networks are studied for small-world-like topologies by finding node betweenness centralities *C*_B_ and calculating the small-world coefficients *σ*_sw_ and *ω*_sw_.^50,52,54^ The betweenness centrality *C*_B_ of a node *i* is found and normalized *via* the NetworkX betweenness_centrality( ) utility and is computed as follows:8
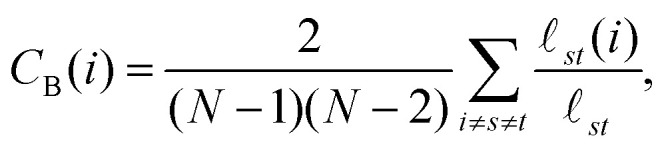
where the pair (*s*, *t*) enumerates over all node pairs in the graph (excluding *i*), ℓ_*st*_ is the total number of shortest paths between *s* and *t*, and ℓ_*st*_(*i*) is the number of shortest paths that flow through node *i*. The normalization coefficient is the inverse of the binomial coefficient 
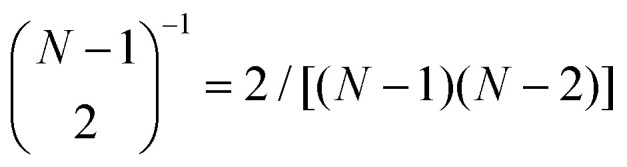
 for a graph with *N* − 1 nodes, enumerating over all combinations of node pairs excluding *i*. Betweenness centralities are normalized to facilitate comparison between graphs of systems of differing sizes, as the summation suggests that it is a metric that scales with the number of nodes *N*.

Graphs are generally considered to have small-world topologies if neighbors of any given node are highly connected to each other, if shortest pathlengths between any given pair of nodes are low, and if the graph is sparse.^50^ Both *σ*_sw_ and *ω*_sw_ serve as estimators of the “small-worldness” of a given graph by comparing its average shortest pathlength *L* = 〈ℓ_min_〉 and its average clustering coefficient *C* to the same quantities *C*_rand_ and *L*_rand_ found for a series of Erdős–Rényi random graphs, and *C*_latt_ and *L*_latt_ for equivalent lattice graphs:9
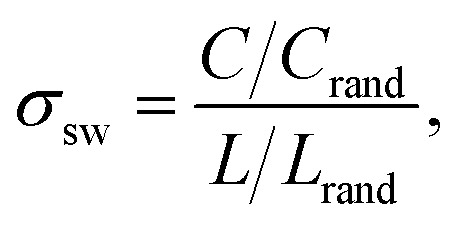
and10
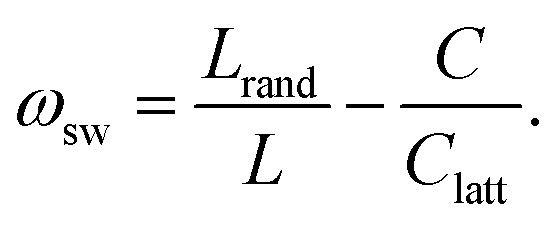


It is commonly recognized that “small-worldness” in a graph corresponds to *σ*_sw_ > 1 and *ω*_sw_ ≈ 0.^52–54^ Small-world network topologies are marked by high clustering and low average pathlengths, *i.e.*, that individual “subcommunities” of nodes exist within the graph that are closely connected to each other and that particular nodes serve as highly-connected hubs bridging each subcommunity together in an efficient manner. The flow of information or impulses within these small-world networks are thus efficient with minimal loss in fidelity.

The betweenness centrality *C*_B_ of a node *i* serves as a metric on its hub-like connectivity, measuring the number of shortest paths between any arbitrary node pair that flows through *i*. For each sampled frame, the ten graph nodes with the highest normalized *C*_B_ are selected as “hubs,” and the ten largest maximal cliques are selected as the “subcommunities” of closely-connected nodes. A maximal clique for any node *i* is defined as the largest fully-connected subgraph containing *i* within the graph of interest; maximal cliques are found *via* the NetworkX find_cliques( ) utility and only reported if fewer than three nodes within the clique are members of an existing reported clique.

### 3.5. Spatial organization within simulated condensates

To study the spatial distribution of topological features from condensate simulations, continuous trajectory samples comprising 20% of total production runs (200 ns of 1 μs in both LCD and YS simulations) are used, and molecular hub and clique statuses are recorded for each frame. Average radial mass density profiles are generated for each simulation to obtain phase interfaces in tandem with data on the radial distribution of hubs and cliques. In each sampled frame, all particle masses and radial distances from the dense-phase center of mass are collected and aggregated, and the mass densities are computed by radial binning. Data on the spatial localization of hubs and cliques are recorded by locating hub and clique molecules within the condensate, computing their centers of mass, determining the radial distances between molecular centers of mass and dense-phase centers of mass, and binning. Sigmoid functions are used to fit each radial mass density profile with the scipy.optimize.curve_fit( ) utility^75^ to quantitatively define interfacial boundaries. The radial bounds of the interface correspond to the radial distances where the mass density is 95% and 5% of the stable dense-phase sigmoid fit value, capturing most of the region of change. Finally, distances in radial distributions are normalized by their corresponding simulation's upper (dilute-phase) interfacial boundary in order to facilitate comparison between systems.

### 3.6. Graph dynamics of the simulated condensate

To understand the time variance of topological organizations, we compute the timescales associated with the presence of hubs and cliques. The same continuous trajectory samples described in the previous Section 3.5. are used. In each frame, the molecular indices corresponding to the ten nodes with the highest betweenness centralities and the molecular indices corresponding to the members of the 10 largest cliques are recorded. The frequency of single-molecule hub or clique status is computed as the number of frames where individual molecules are labeled as hubs or as associated with cliques, respectively. These frequencies are then normalized by the total number of sampled frames in each continuous trajectory sample.

### 3.7. Conformational analysis

To analyze the structural and conformational properties of single polymers in our simulations, we compute single-molecule radii of gyration *R*_g_ and relative shape anisotropies *κ*^2^. These metrics are further employed to quantify changes in IDP conformational properties with respect to molecular connectivities within constructed interaction networks. Entire 1 μs trajectories are sampled at intervals of 20 ns (*i.e.*, every 20th frame). At each sampled frame, graph analyses are performed as described above to compute betweenness centralities *C*_B_ for each molecule. OVITO is used to obtain *R*_g_ values and diagonalized gyration tensors *S* for each individual molecule:11
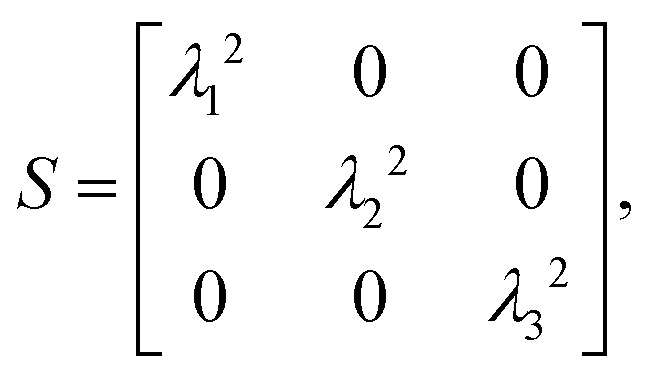
where eigenvalues *λ*_1_, *λ*_2_, *λ*_3_ are the principal components of the molecular gyration tensor. Relative shape anisotropies *κ*^2^ are then obtained *via*12
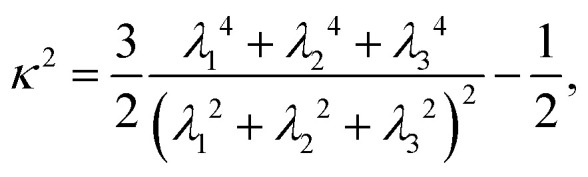
which is bounded between 0 and 1.

### 3.8. Molecular motion through single-molecule displacement

Heterogeneities in molecular movements within condensed phases are studied by measuring single-molecule displacements at 1 ns intervals, the minimum timestep between static frames in our LCD and YS simulations. As in our conformational analyses, we sample frames across trajectories of length 1 μs at intervals of 20 ns. For each of these frames, sampled at some timestep *t*, we compute the center of mass **r**_*i*,COM_(*t*) of each molecule *i*. The magnitudes of “instantaneous” displacements |Δ**r**_*i*_| are obtained by averaging the differentials of the **r**_*i*,COM_ from frames 1 ns before and after the sampled frame at *t*, *i.e.*, by computing **r**_*i*,COM_(*t* − 1) and **r**_*i*,COM_(*t* + 1), respectively:13
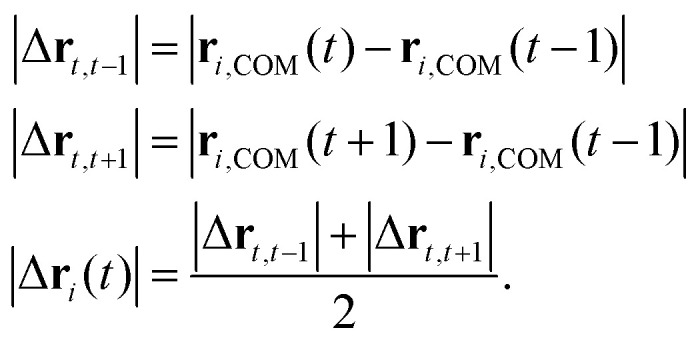


Molecular displacements |Δ**r**| are also normalized by the length of the corresponding polymer's linear chain conformation to obtain |Δ̃**r**| for comparison. Graph analyses are then performed to relate single-molecule displacement to molecular connectivity and topological status.

### 3.9. Surface tension calculation

Surface tension between two phases of different densities is usually described by the Kirkwood–Buff formalism,^76,77^ considering that the presence of an interface provides anisotropy to the overall pressure tensor. Thus, following the definition of mechanical equilibrium, the surface tension is given by14
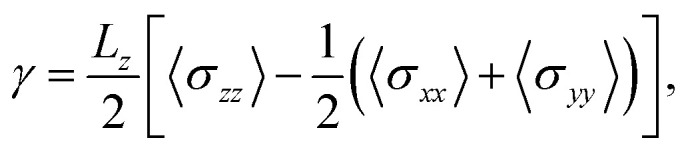
where *L*_*z*_ is the length of the slab, and 〈*σ*_*ii*_〉 are the ensemble averages of the diagonal components of the pressure tensor.

However, the formalism is built upon the assumption of a sharp interface, which breaks down as a condensate's critical temperature is approached. At that limit, the interface becomes more diffuse and harder to define, yielding incorrect surface tension estimations through the Kirkwood–Buff formalism. To overcome this difficulty, we employ a stress-profile method calculated from per-atom stresses using the virial force contribution equation.^78^ This derivation is possible because anisotropy of the diagonal components of the pressure tensors are found at the dense-dilute interface:15
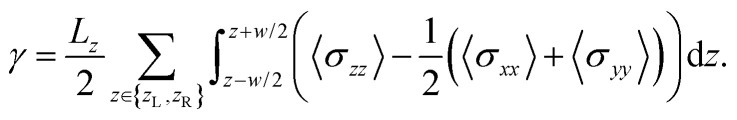
Here *z*_L_, *z*_R_ correspond to the left and right interfaces of the dense phase in the slab geometry, with an equal thickness *w*.

### 3.10. Computation of effective packing densities

To determine whether the dynamic and structural effects observed in this work can be partially attributed to broader polymer-physics considerations such as packing and crowding within condensate volumes, we estimate the effective packing density *ϕ*_rel_ of each simulated condensate relative to the overlap concentration c*, or the concentration at which polymers begin to significantly interpenetrate. We define the effective packing density *ϕ*_rel_ as16
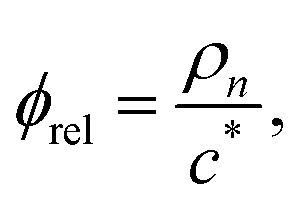
where *ρ*_*n*_ is the measured number density of monomers in a sampled volume within the dense phase, and the overlap concentration c* is the number density of monomers within the excluded volume of a dilute single chain.^61,62^ In other words, we define c* as17
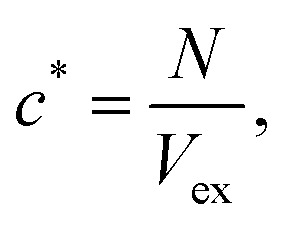
where *N* is the number of monomers per chain and *V*_ex_ is the dilute single-chain excluded volume. We estimate the single-chain excluded volume by equilibrating single chains in large simulation cells at *T* = 0.90*T*_c_ and sampling their conformations. We then compute *V*_ex_ for each sample by summing the volumes of all monomers *i* in the chain of length *N* and subtracting the volumes of the intersections between contiguous (bonded) monomers:18
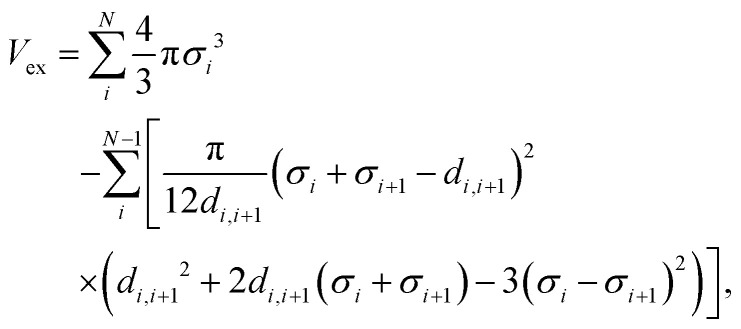
where *σ*_*i*_ corresponds to the radius of the spherical monomer *i*, obtained from the parameters of the Mpipi model,^42^*σ*_*i*+1_ corresponds to the radius of the adjacent monomer *i* + 1, and *d*_*i*,*i*+1_ refers to the distance between the centers of two contiguous (bonded) monomers *i* and *i* + 1. The first term in *V*_ex_ sums all the spherical monomeric volumes comprising a single chain, and the second term in *V*_ex_ subtracts the volume of the intersection between two adjacent monomers for all *N* − 1 pairs of adjacent/bonded monomers. The average of all sampled *V*_ex_ is returned for each LCD and YS sequence along with the associated standard error.

To measure the monomeric number density *ρ*_*n*_ inside each condensate, we construct a spherical volume with a radius equal to the distance between the dense-phase center of mass and the center of the located interface (see Methods Section 3.5, “Spatial organization within simulated condensates”, for details; alternatively, see Fig. 3b for a visualization of radial interface location). As in the preceding analyses of conformation and dynamics, entire 1 μs trajectories are sampled at intervals of 20 ns for both LCD and YS simulations. For each sampled frame, we center the constructed spherical volume on the dense-phase center of mass, record the number of monomeric units present within this volume, and then divide the monomer count by the sphere volume. Monomer number densities are averaged over all sampled frames for each simulation; this average *ρ*_*n*_ is returned for each LCD and YS sequence along with the associated standard error.

Based on this construction, an effective packing density *ϕ*_rel_ < 1 indicates that the condensate is dilute-like, *i.e.*, that packing in the dense phase is below the threshold after which polymers begin to overlap. *ϕ*_rel_ ≈ 1 indicates that the number density of monomers in the dense phase is roughly equivalent to the number density of monomers within the excluded volume of a single chain, *i.e.*, that the chains in the dense phase are beginning to overlap. *ϕ*_rel_ > 1 indicates that the system is in the semi-dilute or concentrated regime, where the substantial packing and overlapping of polymers strongly influences the macroscopic features of the dense phase.

## Author contributions

D. T., D. A. and J. A. J. conceived the study. D. T., D. A. and P. L. G. performed simulations and analyses. D. T. and D. A. curated and visualized data. D. T., D. A., P. L. G. and J. A. J. wrote and edited the manuscript. D. T. and J. A. J. acquired research funding. D. A. and J. A. J. supervised the research.

## Conflicts of interest

The authors declare no conflicts of interest.

## Supplementary Material

SM-021-D5SM00740B-s001

## Data Availability

Supplementary information is available. See DOI: https://doi.org/10.1039/d5sm00740b. The data supporting the findings in this study, as well as sample simulation input and output files, are available at the Joseph Group GitHub repository: https://github.com/josephresearch/LCD_Network.
