# Supplementary material for: Biomolecular condensate microstructure is invariant to sequence-encoded molecular and macroscopic properties
Source: Soft Matter. 2025 Sep 23;21(45):8635–54. doi: 10.1039/d5sm00740b (PMC12455476; doi:10.1039/d5sm00740b)
Supplement: SM-021-D5SM00740B-s001 [file SM-021-D5SM00740B-s001.pdf]

Supplementary Information (SI) for Soft Matter.
This journal is © The Royal Society of Chemistry 2025

Supporting information for:
Biomolecular condensate microstructure is invariant to sequence-encoded molecular and macroscopic properties

Daniel Tan,[1,*] Dilimulati Aierken,[1,2,*] Pablo L. Garcia,[1] and Jerelle A. Joseph[1,2,†]
[1]Department of Chemical and Biological Engineering, Princeton University, Princeton, NJ 08544, USA
[2]Omenn–Darling Bioengineering Institute, Princeton University, Princeton, NJ 08544, USA
(Dated: August 21, 2025)

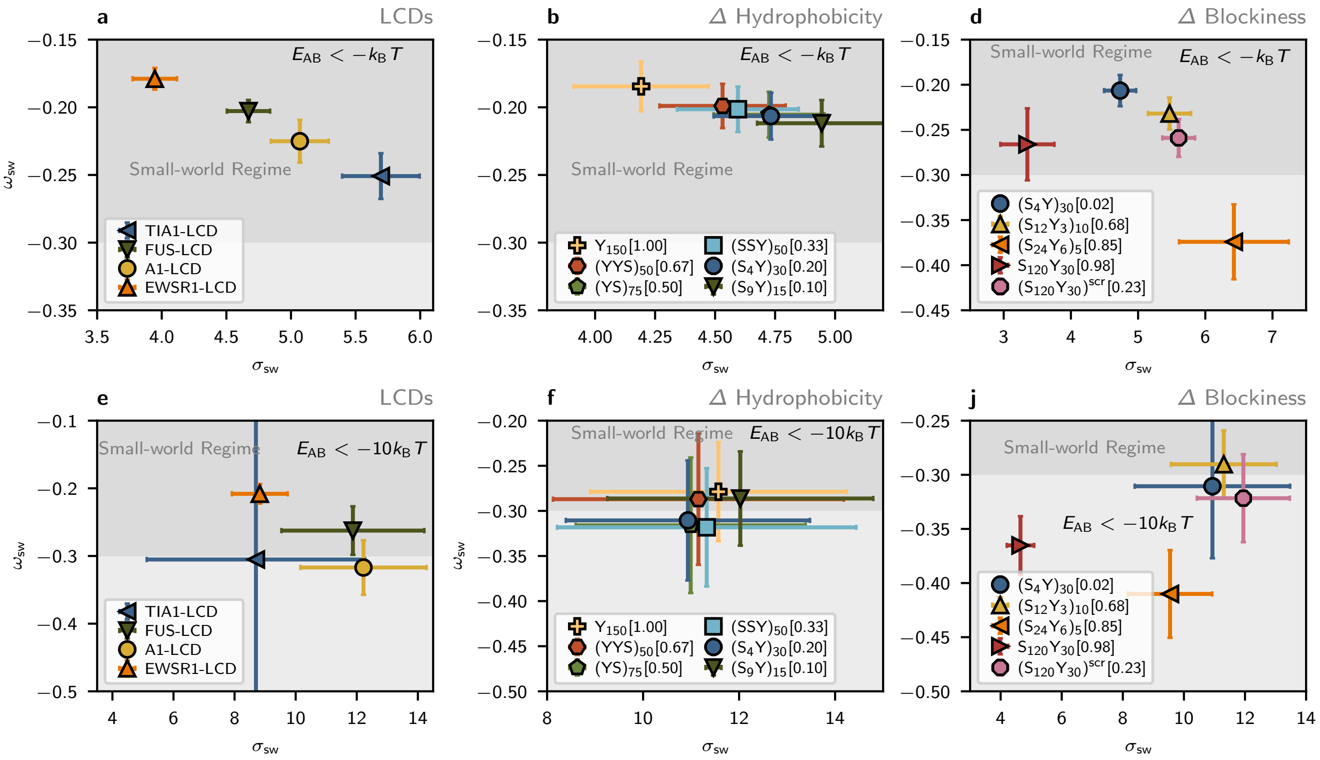

FIG. S1. **a,e** Small-world parameters σ_{sw} and ω_{sw} for single-component LCD condensates with energy threshold $E_{AB} < -k_B T$ and $E_{AB} < -10k_B T$, respectively. **b,f** Small-world parameters σ_{sw} and ω_{sw} for condensates formed by YS sequences with varying hydrophobicity with energy threshold $E_{AB} < -k_B T$ and $E_{AB} < -10k_B T$, respectively. **d,j** Small-world parameters σ_{sw} and ω_{sw} for condensates formed by YS sequences with $f_Y = 0.2$ and varying patterning with energy threshold $E_{AB} < -k_B T$ and $E_{AB} < -10k_B T$, respectively.

* These authors contributed equally
† Corresponding author: jerellejoseph@princeton.edu

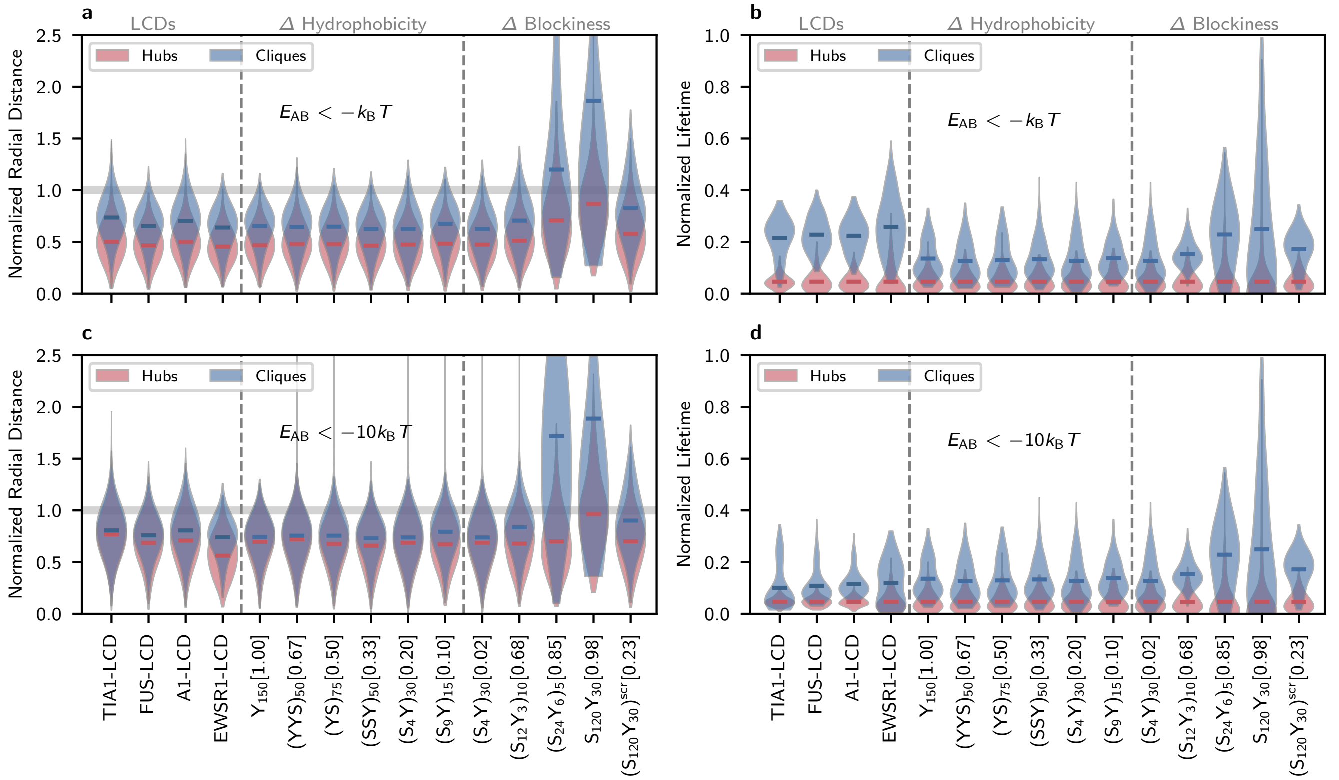

FIG. S2. **a,c** The distribution of hubs and cliques for all simulated sequences in terms of normalized radial distance from the condensate center of mass to interface with energy threshold $E_{AB} < -k_B T$ and $E_{AB} < -10k_B T$, respectively. Distances are normalized by the radial location of the interface in each simulation, overlaid in grey. YS sequences with varying hydrophobicity and conserved patterning are labeled with their hydrophobic fraction f_Y. YS sequences with varying patterning and conserved composition are labeled with their sequence blockiness f_B, described in the Methods. **b,d** Normalized lifetimes of hub molecules and clique molecules are shown for all simulated sequences with energy threshold $E_{AB} < -k_B T$ and $E_{AB} < -10k_B T$, respectively. Lifetimes are normalized by 200 ns, the length of the continuous trajectory sample used to generate these measurements. YS variants are labeled with f_Y (Δ hydrophobicity) and f_B (Δ blockiness) as described in (a,c).

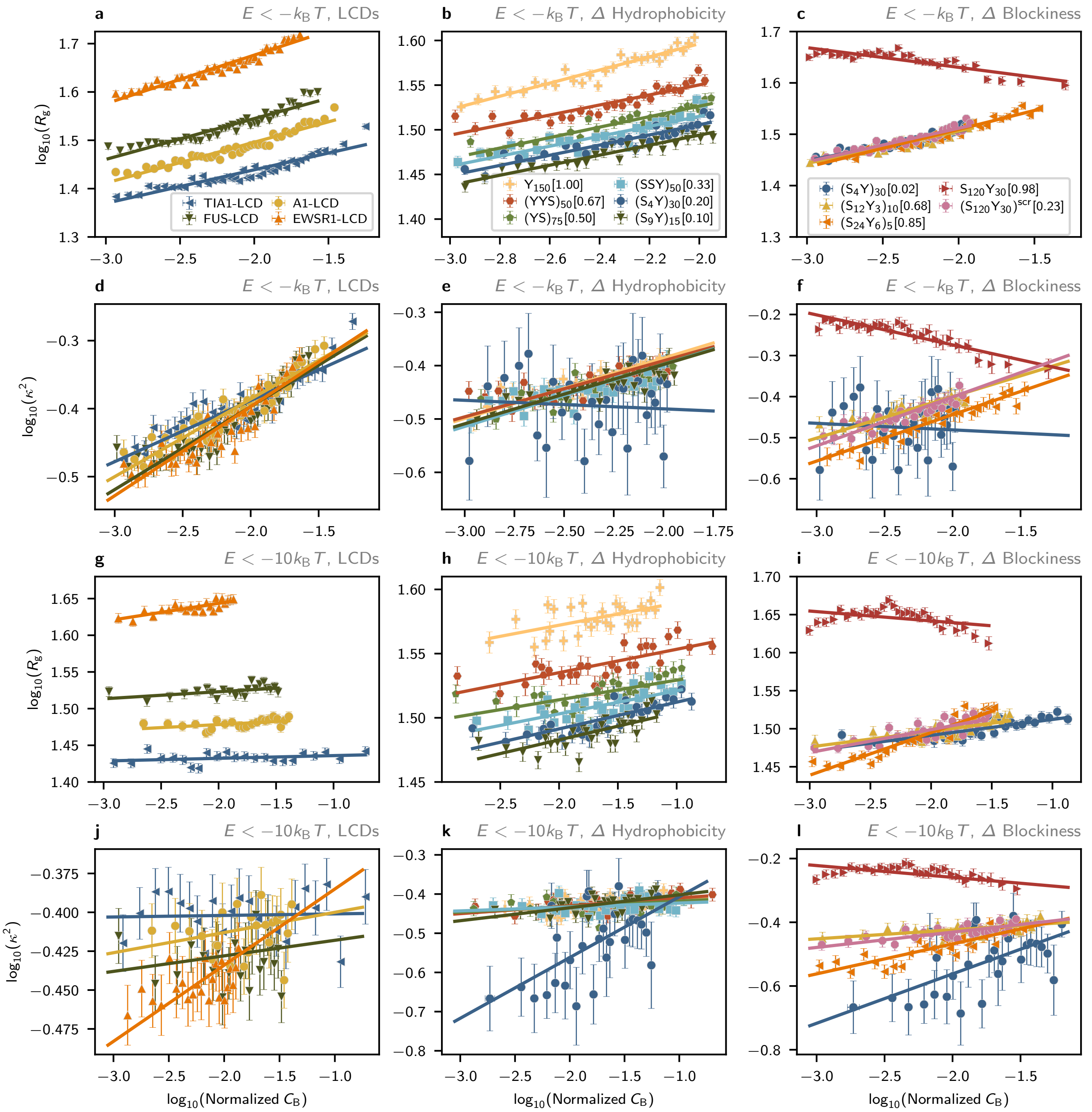

FIG. S3. **a, b, c, g, h, i** Possible power-law relations between the betweenness centrality C_{B} and radius of gyration R_{g}, indicated by linear fits in $\log_{10}$–$\log_{10}$ space with energy threshold $E_{\mathrm{AB}} < -k_{\mathrm{B}}T$ and $E_{\mathrm{AB}} < -10k_{\mathrm{B}}T$, respectively.. **d, e, f, j, k, l** Possible power-law relations between the betweenness centrality C_{B} and relative shape anisotropy κ^2 with energy threshold $E_{\mathrm{AB}} < -k_{\mathrm{B}}T$ and $E_{\mathrm{AB}} < -10k_{\mathrm{B}}T$, respectively.

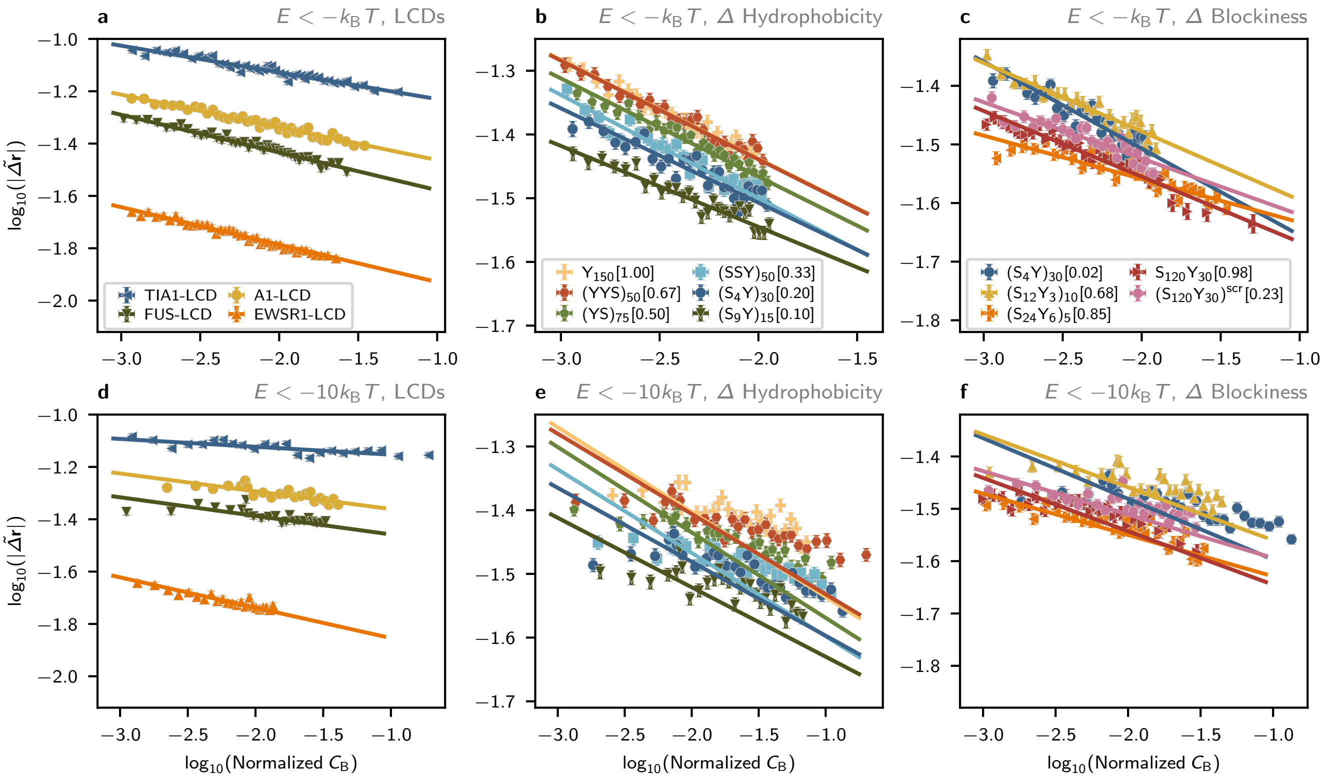

FIG. S4. **a, b, c** Normalized instantaneous displacements $|\tilde{\Delta}\mathbf{r}|$ of single chains in condensed phases exhibit strong negative correlations with node betweenness centrality C_B within molecular graphs with energy threshold $E_{AB} < -k_B T$. **d, e, f** Normalized instantaneous displacements $|\tilde{\Delta}\mathbf{r}|$ of single chains in condensed phases exhibit strong negative correlations with node betweenness centrality C_B within molecular graphs with energy threshold $E_{AB} < -10 k_B T$.

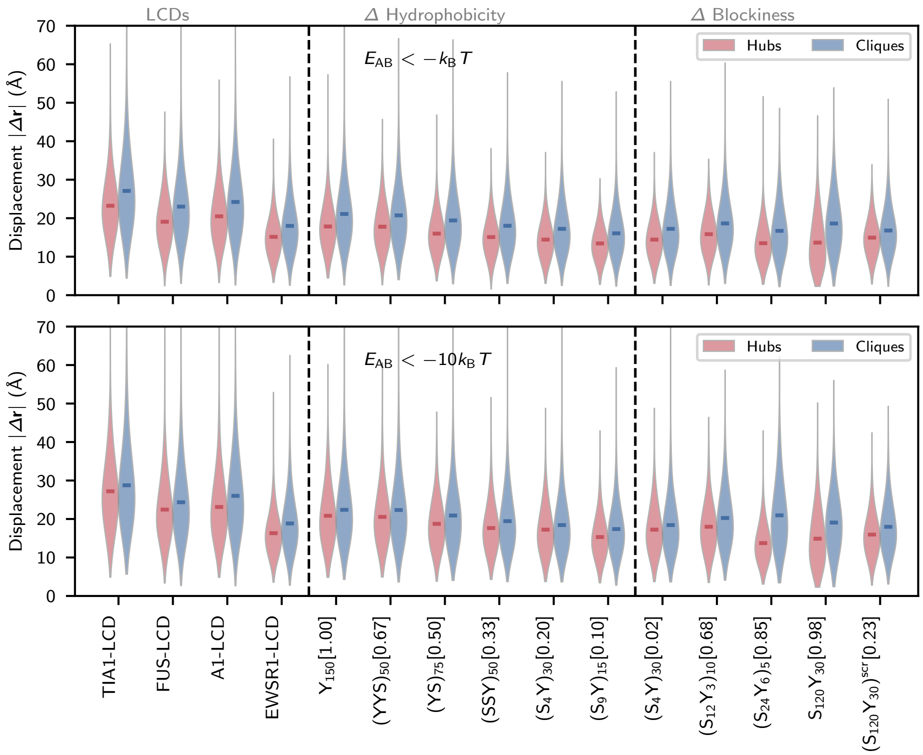

FIG. S5. **a, b** Displacements within consistent time intervals are compared for hub molecules and clique molecules with energy threshold $E_{AB} < -k_B T$ and $E_{AB} < -10k_B T$ (top and bottom, respectively).

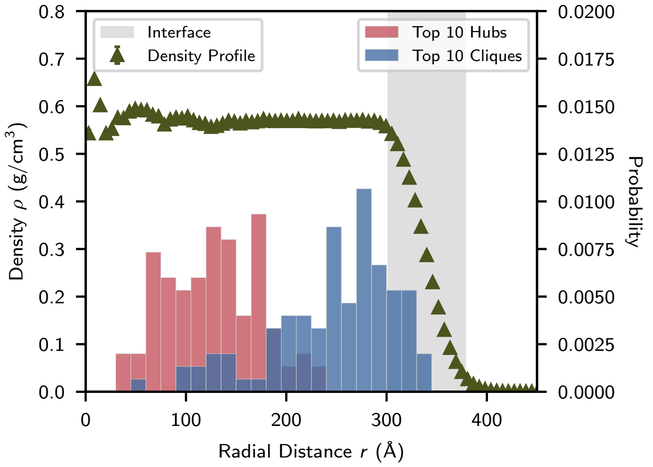

FIG. S6. Spatial distributions of hubs and cliques in a single-component FUS-LCD condensate containing 3375 chains at $T = 300$K. A radial mass density profile is shown in green triangles, and the phase interface is overlaid in grey. Consistent with results from simulations of smaller systems, clique molecules are closer to the condensate interface than hub molecules.

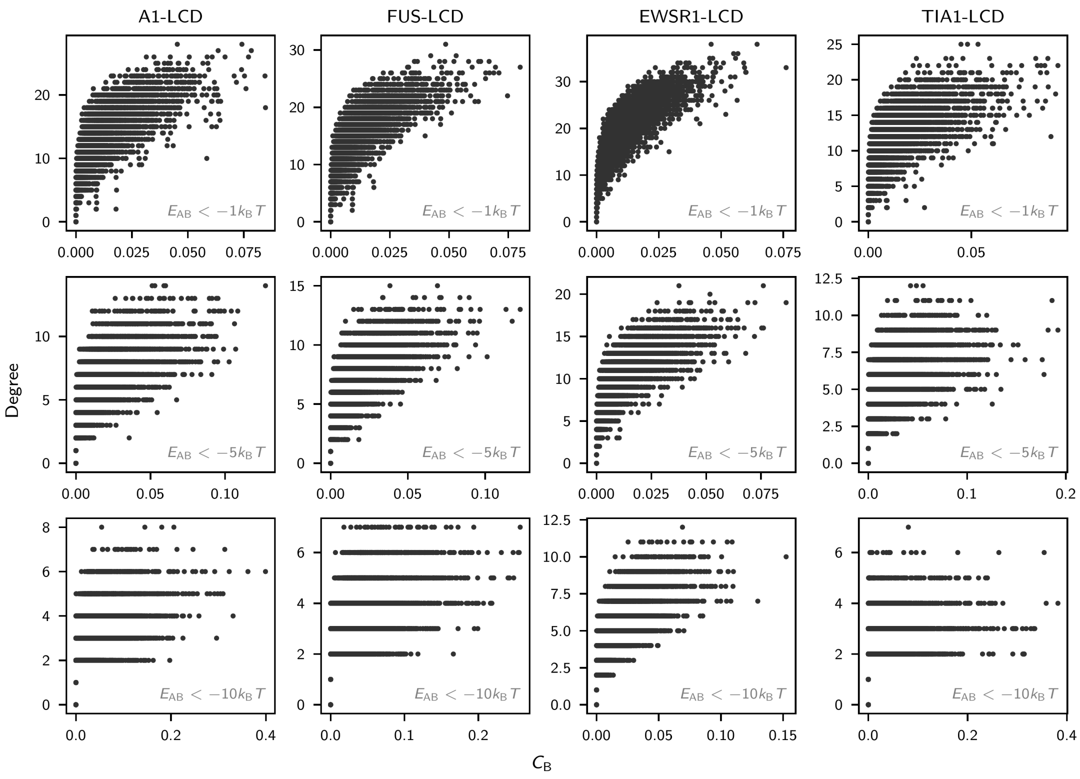

FIG. S7. Correlation between node degree and normalized betweenness centrality values C_B in interaction networks constructed for LCD simulations at $T = 0.90T_c$. Graphs with edges assigned using the $E_{AB} < -1k_BT$ threshold are shown in the top row, $E_{AB} < -5k_BT$ in the center row, and $E_{AB} < -10k_BT$ in the bottom row. The positive correlation between node degree and betweenness centrality reflects the tendency of a node to connect more graph substructures when it connects many nodes, but the spread in the data indicate that a high node degree is not required for high betweenness centrality and vice versa.

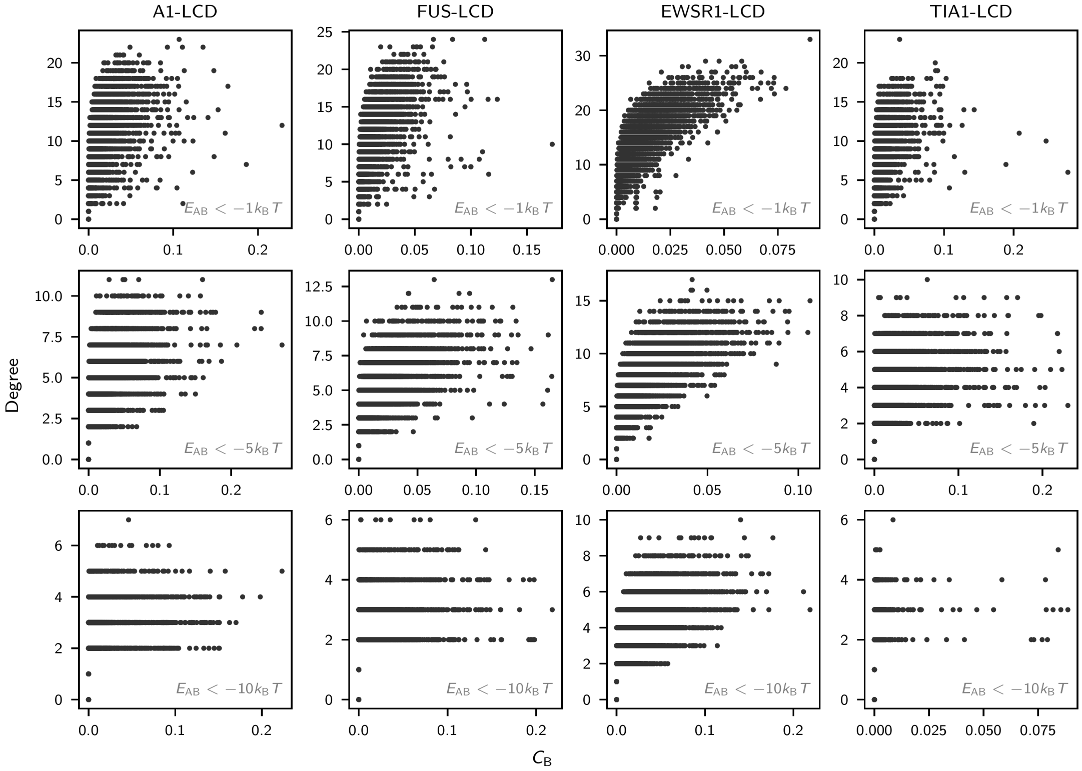

FIG. S8. Correlation between node degree and normalized betweenness centrality values C_B in interaction networks constructed for LCD simulations at $T = 0.95T_c$. Graphs with edges assigned using the $E_{AB} < -1k_B T$ threshold are shown in the top row, $E_{AB} < -5k_B T$ in the center row, and $E_{AB} < -10k_B T$ in the bottom row.

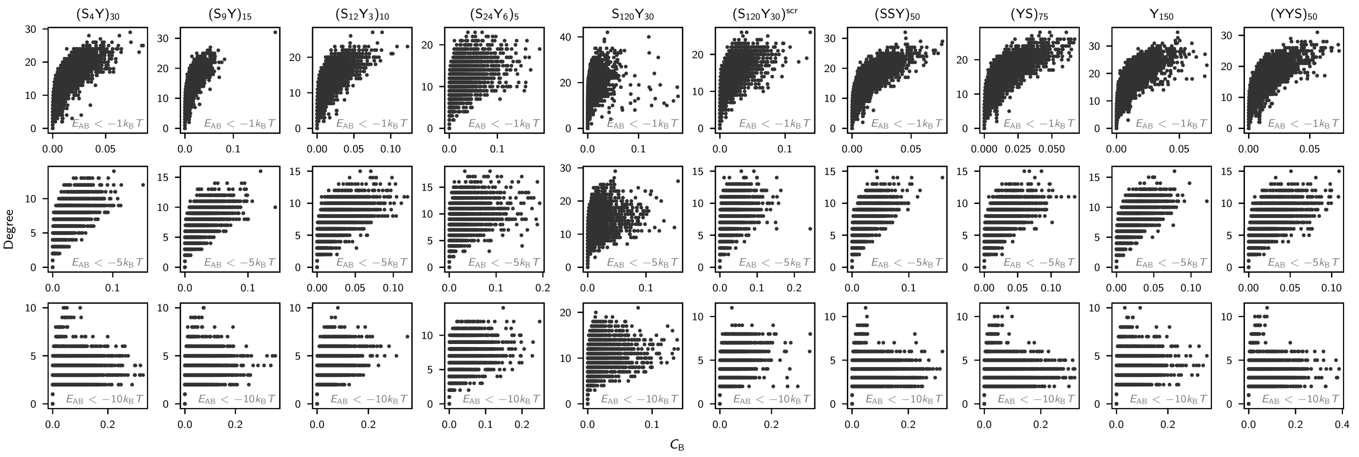

FIG. S9. Correlation between node degree and normalized betweenness centrality values C_{B} in interaction networks constructed for simulations of YS variants at $T = 0.90T_{\mathrm{c}}$. Graphs with edges assigned using the $E_{\mathrm{AB}} < -1k_{\mathrm{B}}T$ threshold are shown in the top row, $E_{\mathrm{AB}} < -5k_{\mathrm{B}}T$ in the center row, and $E_{\mathrm{AB}} < -10k_{\mathrm{B}}T$ in the bottom row. Though a positive trend is visible, the spread in the data indicate that high node degrees are not required for high betweenness centralities and vice versa.

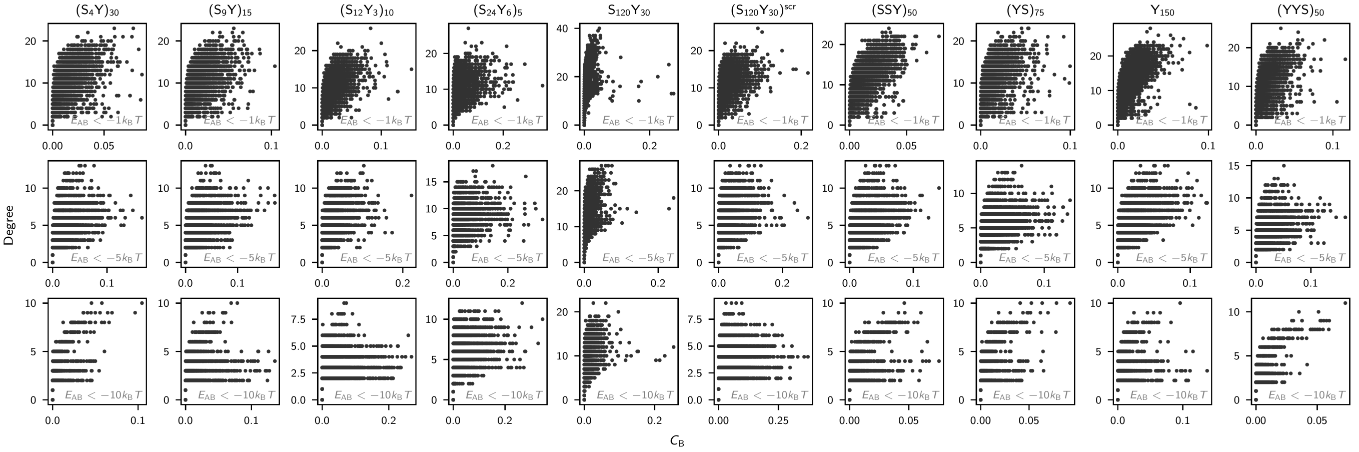

FIG. S10. Correlation between node degree and normalized betweenness centrality values C_{B} in interaction networks constructed for simulations of YS variants at $T = 0.95T_{\mathrm{c}}$. Graphs with edges assigned using the $E_{\mathrm{AB}} < -1k_{\mathrm{B}}T$ threshold are shown in the top row, $E_{\mathrm{AB}} < -5k_{\mathrm{B}}T$ in the center row, and $E_{\mathrm{AB}} < -10k_{\mathrm{B}}T$ in the bottom row.

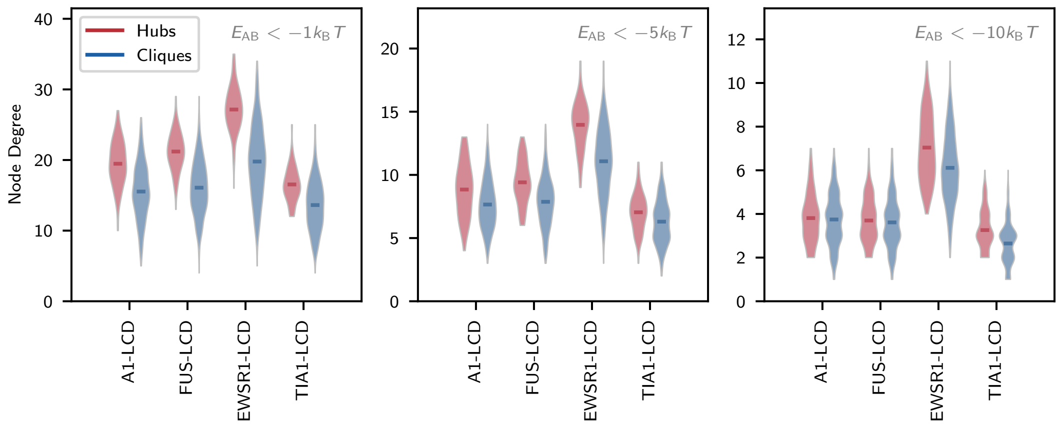

FIG. S11. Node degree distributions recorded for hub molecules (colored red) and clique molecules (colored blue), shown for simulations of LCD sequences at $T = 0.90\,T_c$. On average, hub molecules assume a higher degree (interact with more associative partners) than clique molecules, as expected. However, their overlapping distributions indicate that qualities of mesoscale organization, such as hublike or clique-like character, are inaccessible by analyzing degree alone.

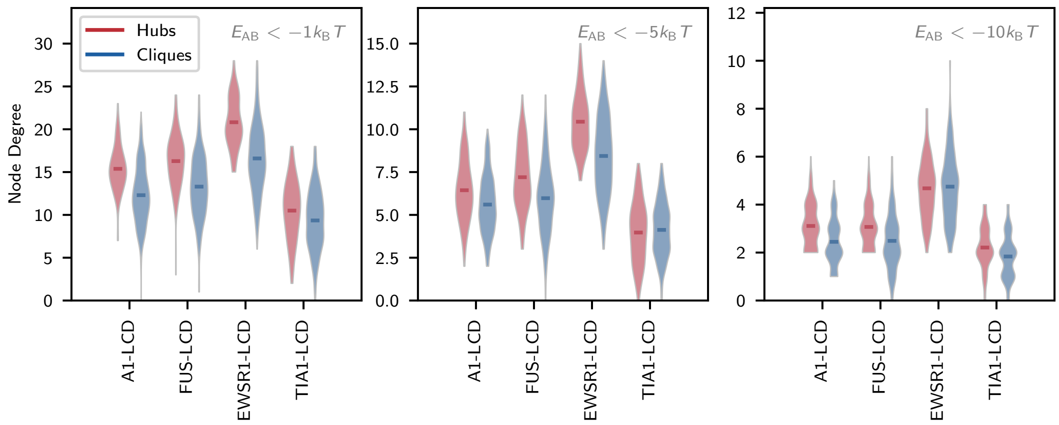

FIG. S12. Node degree distributions recorded for hub molecules (colored red) and clique molecules (colored blue), shown for simulations of LCD sequences at $T = 0.95\,T_c$.

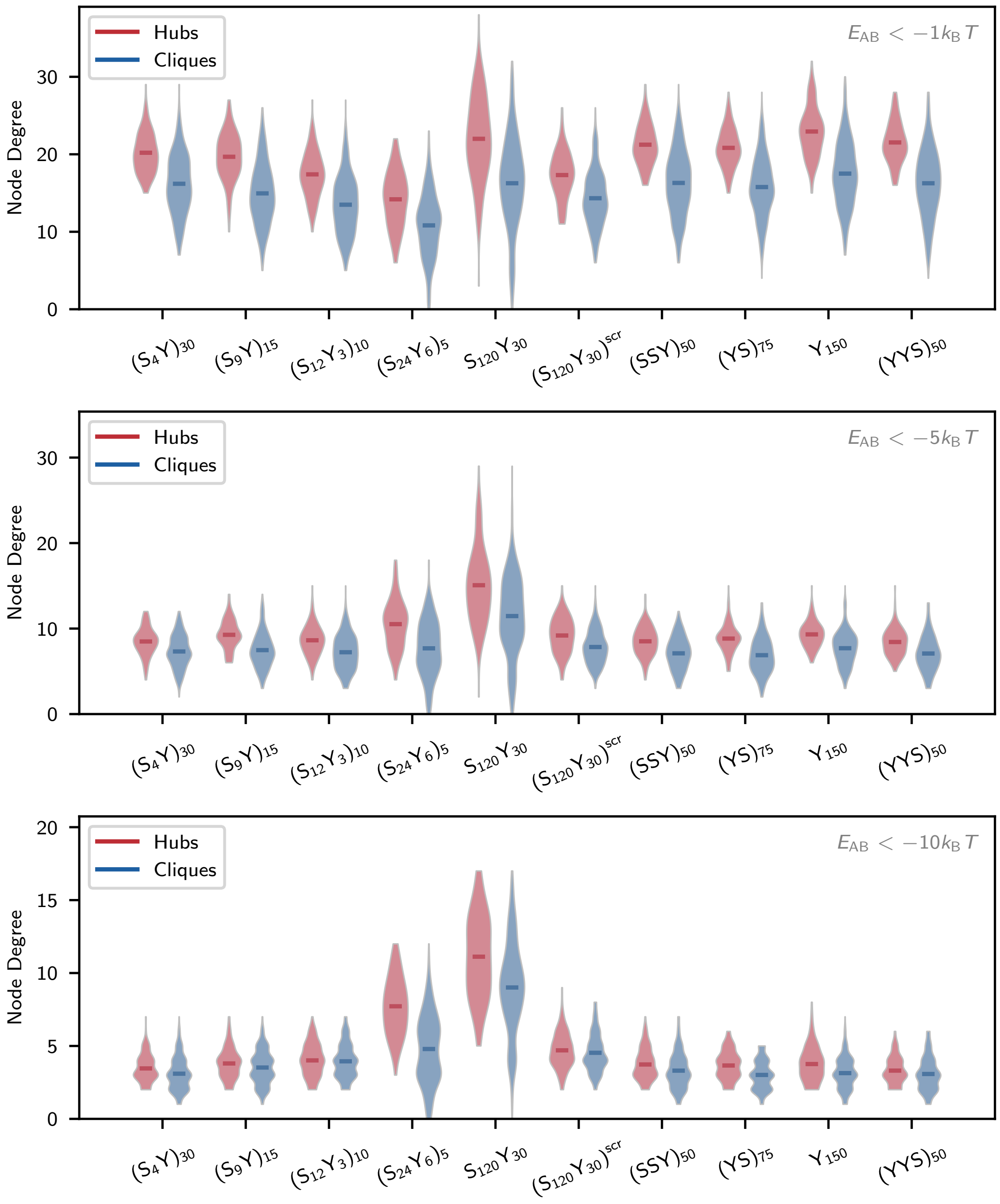

FIG. S13. Node degree distributions recorded for hub molecules (colored red) and clique molecules (colored blue), shown for simulations of YS variant sequences at $T = 0.90\,T_c$. On average, hub molecules assume a higher degree (interact with more associative partners) than clique molecules, as expected. However, their overlapping distributions indicate that qualities of mesoscale organization, such as hublike or clique-like character, are inaccessible by analyzing degree alone.

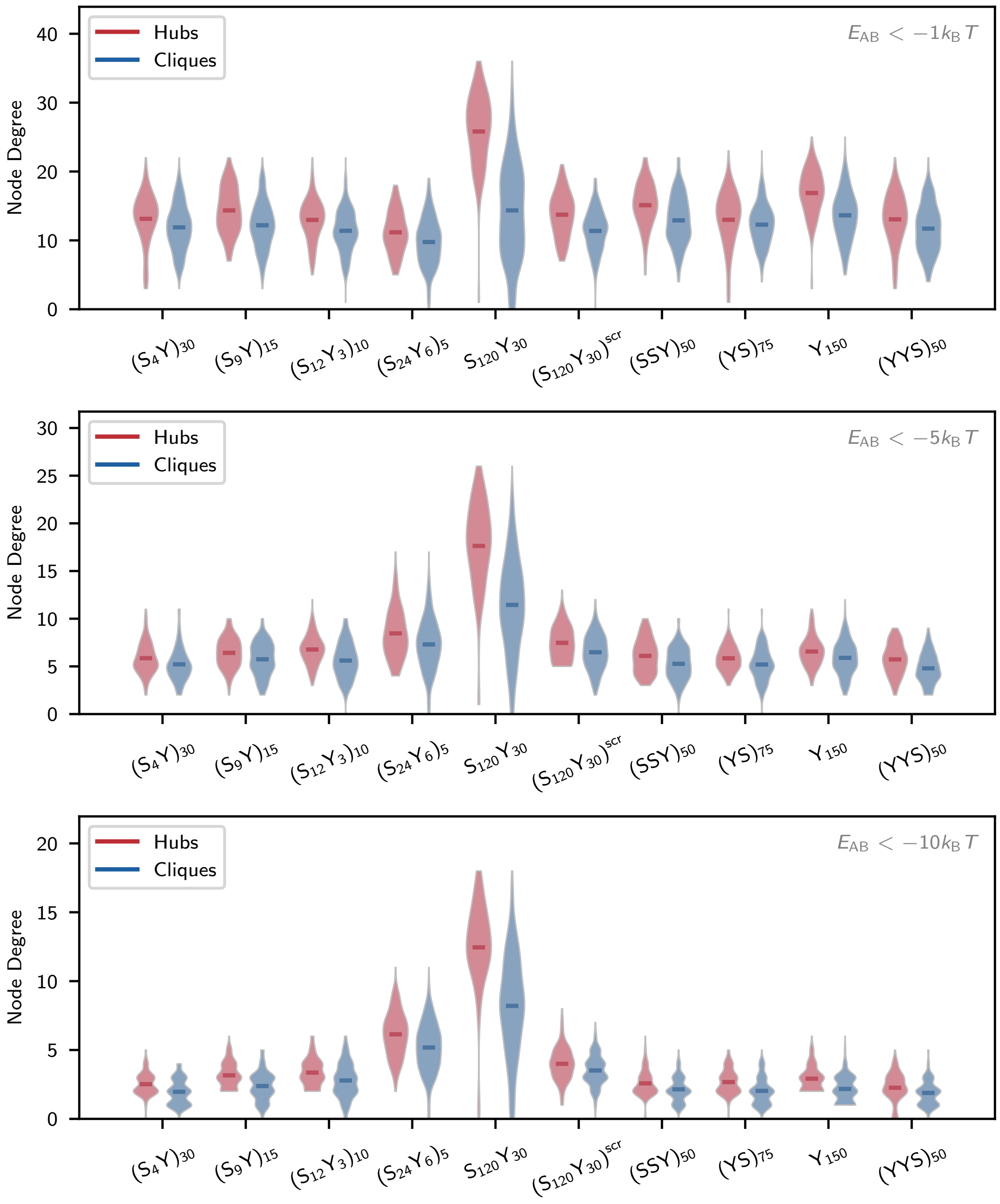

FIG. S14. Node degree distributions recorded for hub molecules (colored red) and clique molecules (colored blue), shown for simulations of YS variant sequences at $T = 0.90\,T_c$.

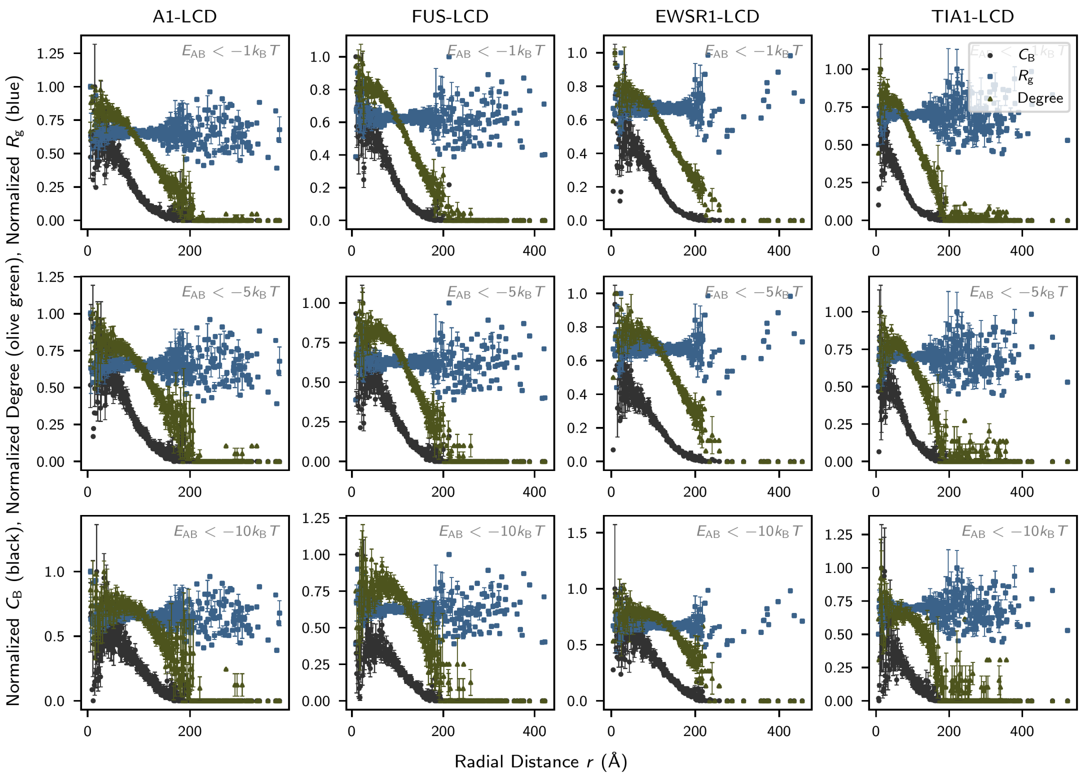

FIG. S15. Average dependence of node betweenness centrality (black circles), node degree (green triangles), and chain radius of gyration (blue squares) on the radial distance from the condensate center, shown for simulations of LCD sequences at $T = 0.90T_c$. Data for graphs with edges assigned using the $E_{AB} < -1k_BT$ threshold are shown in the top row, $E_{AB} < -5k_BT$ in the center row, and $E_{AB} < -10k_BT$ in the bottom row. C_B, degree, and R_g (Å) are normalized by their maximal values in each subplot to aid visualization. Maximal values for each metric (i.e., inverse normalization coefficients) are presented in Table S1.

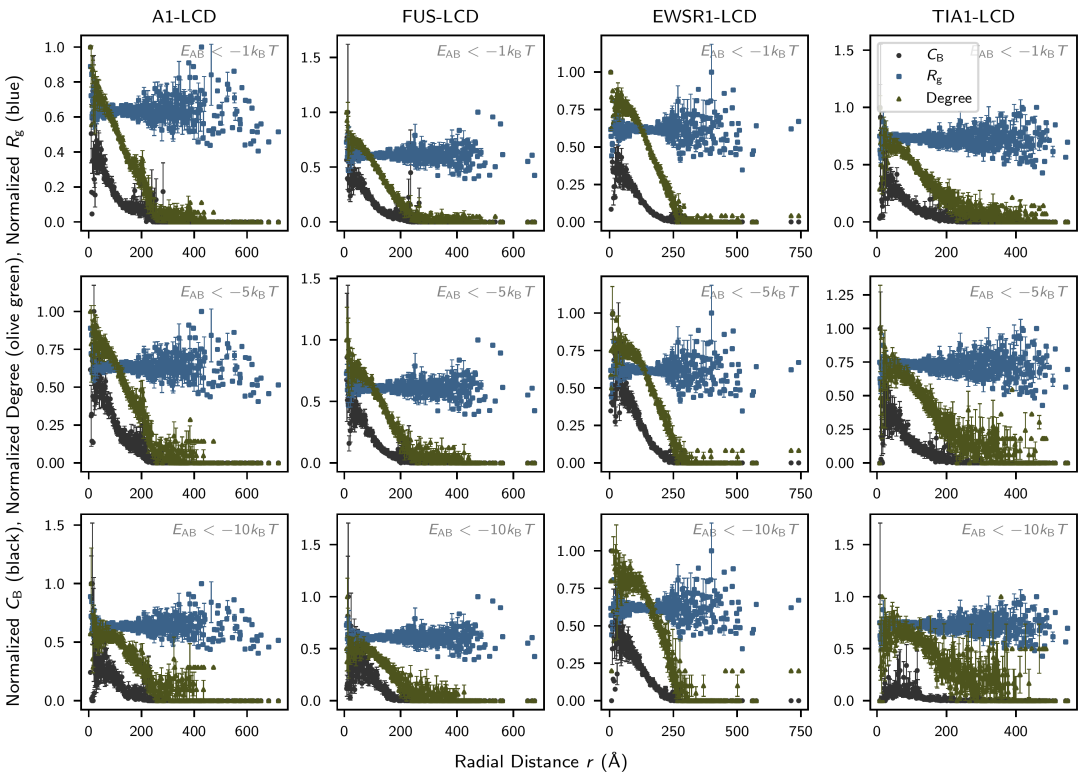

FIG. S16. Average dependence of node betweenness centrality (black circles), node degree (green triangles), and chain radius of gyration (blue squares) on the radial distance from the condensate center, shown for simulations of LCD sequences at $T = 0.95T_c$. Data for graphs with edges assigned using the $E_{AB} < -1k_BT$ threshold are shown in the top row, $E_{AB} < -5k_BT$ in the center row, and $E_{AB} < -10k_BT$ in the bottom row. Values are normalized by their maximal value in each subplot to aid visualization. Maximal values for each metric (i.e., inverse normalization coefficients) are presented in Table S1.

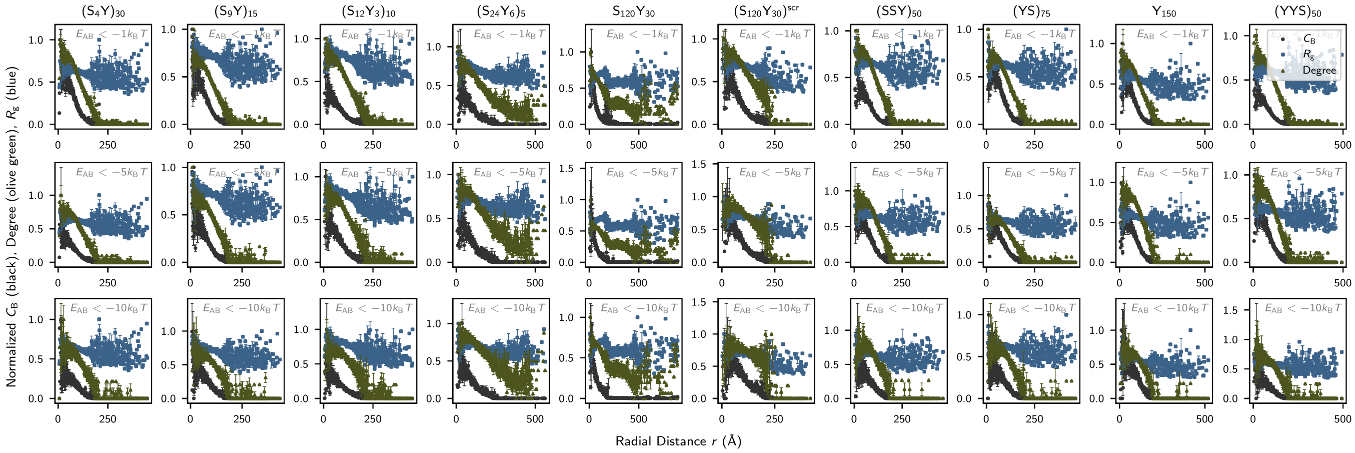

FIG. S17. Average dependence of node betweenness centrality (black circles), node degree (green triangles), and chain radius of gyration (blue squares) on the radial distance from the condensate center, shown for simulations of YS variants at $T = 0.90T_c$. Data for graphs with edges assigned using the $E_{AB} < -1k_BT$ threshold are shown in the top row, $E_{AB} < -5k_BT$ in the center row, and $E_{AB} < -10k_BT$ in the bottom row. Values are normalized by their maximal value in each subplot to aid visualization. Maximal values for each metric (i.e., inverse normalization coefficients) are presented in Table S2.

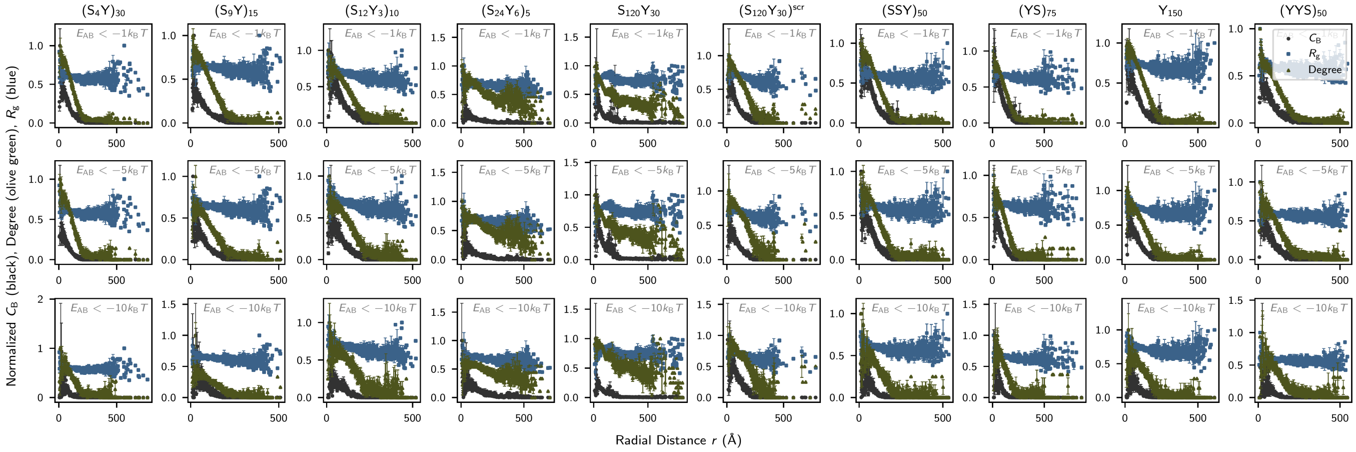

FIG. S18. Average dependence of node betweenness centrality (black circles), node degree (green triangles), and chain radius of gyration (blue squares) on the radial distance from the condensate center, shown for simulations of YS variants at $T = 0.95T_c$. Data for graphs with edges assigned using the $E_{AB} < -1k_BT$ threshold are shown in the top row, $E_{AB} < -5k_BT$ in the center row, and $E_{AB} < -10k_BT$ in the bottom row. Values are normalized by their maximal value in each subplot to aid visualization. Maximal values for each metric (i.e., inverse normalization coefficients) are presented in Table S3.

Sequence	Temperature $/T_c$	Energy Threshold $/-k_B T$	Max C_B	Max Degree	Max R_g /Å
A1-LCD	0.90	1	0.0419	21.0	45.8911
A1-LCD	0.90	5	0.0550	9.5	45.8911
A1-LCD	0.90	10	0.0992	4.0769	45.8911
A1-LCD	0.95	1	0.0709	18.0	47.0154
A1-LCD	0.95	5	0.0666	7.0	47.0154
A1-LCD	0.95	10	0.0416	3.5	47.0154
FUS-LCD	0.90	1	0.0420	23.3	53.8668
FUS-LCD	0.90	5	0.0566	10.0	53.8668
FUS-LCD	0.90	10	0.1214	4.0	53.8668
FUS-LCD	0.95	1	0.0599	20.0	55.7586
FUS-LCD	0.95	5	0.0823	8.0	55.7586
FUS-LCD	0.95	10	0.0391	4.0	55.7586
EWSR1-LCD	0.90	1	0.0446	32.0	66.4045
EWSR1-LCD	0.90	5	0.0614	16.0	66.4045
EWSR1-LCD	0.90	10	0.0574	7.5	66.4045
EWSR1-LCD	0.95	1	0.0720	24.0	73.2549
EWSR1-LCD	0.95	5	0.0586	12.0	73.2549
EWSR1-LCD	0.95	10	0.1088	5.0	73.2549
TIA1-LCD	0.90	1	0.0618	18.0	38.0820
TIA1-LCD	0.90	5	0.0785	7.3	38.0820
TIA1-LCD	0.90	10	0.0742	3.25	38.0820
TIA1-LCD	0.95	1	0.0510	14.0	35.9690
TIA1-LCD	0.95	5	0.0716	5.5	35.9690
TIA1-LCD	0.95	10	0.0057	2.0	35.9690

TABLE S1. Maximal measurement values for node betweenness centrality C_B (unitless), node degree (unitless), and single-chain radius of gyration R_g (Å) from simulations of LCD sequences. Tabulated values are used to normalize measurements of C_B, degree, and R_g in visualizing their relationship to molecular distance from the condensate center of mass in Figs. S15 and S16.

Sequence	Temperature $/T_c$	Energy Threshold $/-k_BT$	Max C_B	Max Degree	Max R_g /Å
$(S_4Y)_{30}$	0.90	1	0.0404	22.0	50.5730
$(S_4Y)_{30}$	0.90	5	0.094	10.0	50.5730
$(S_4Y)_{30}$	0.90	10	0.1672	4.6	50.5730
$(S_9Y)_{15}$	0.90	1	0.0519	26.0	42.9463
$(S_9Y)_{15}$	0.90	5	0.0810	12.0	42.9463
$(S_9Y)_{15}$	0.90	10	0.1270	4.8	42.9463
$(S_{12}Y_3)_{10}$	0.90	1	0.0777	19.0	39.9003
$(S_{12}Y_3)_{10}$	0.90	5	0.0762	10.0	39.9003
$(S_{12}Y_3)_{10}$	0.90	10	0.1622	5.0	39.9003
$(S_{24}Y_6)_5$	0.90	1	0.0743	16.0	43.5978
$(S_{24}Y_6)_5$	0.90	5	0.0770	11.0	43.5978
$(S_{24}Y_6)_5$	0.90	10	0.0916	8.0	43.5978
$S_{120}Y_{30}$	0.90	1	0.0335	28.0	64.8506
$S_{120}Y_{30}$	0.90	5	0.0409	22.5	64.8506
$S_{120}Y_{30}$	0.90	10	0.0476	15.0	64.8506
$(S_{120}Y_{30})^{scr}$	0.90	1	0.0504	18.5	46.9385
$(S_{120}Y_{30})^{scr}$	0.90	5	0.0534	9.2	46.9385
$(S_{120}Y_{30})^{scr}$	0.90	10	0.0750	5.0	46.9385
$(SSY)_{50}$	0.90	1	0.0601	24.0	50.2288
$(SSY)_{50}$	0.90	5	0.0535	9.0	50.2288
$(SSY)_{50}$	0.90	10	0.1317	3.8	50.2288
$(YS)_{75}$	0.90	1	0.0427	26.0	53.5352
$(YS)_{75}$	0.90	5	0.0641	12.0	53.5352
$(YS)_{75}$	0.90	10	0.1508	4.8	53.5352
Y_{150}	0.90	1	0.0401	28.0	68.4950
Y_{150}	0.90	5	0.0545	10.0	68.4950
Y_{150}	0.90	10	0.0976	4.5	68.4950
$(YYS)_{50}$	0.90	1	0.0740	25.0	59.1013
$(YYS)_{50}$	0.90	5	0.0601	9.1	59.1013
$(YYS)_{50}$	0.90	10	0.1495	4.0	59.1013

TABLE S2. Maximal measurement values for node betweenness centrality C_B (unitless), node degree (unitless), and single-chain radius of gyration R_g (Å) from simulations of YS variant sequences at $T = 0.90T_c$. Tabulated values are used to normalize measurements of C_B, degree, and R_g in visualizing their relationship to molecular distance from the condensate center of mass in Fig. S17.

Sequence	Temperature $/T_c$	Energy Threshold $/-k_BT$	Max C_B	Max Degree	Max R_g /Å
$(S_4Y)_{30}$	0.95	1	0.0485	16.5	52.8052
$(S_4Y)_{30}$	0.95	5	0.1055	7.0	52.8052
$(S_4Y)_{30}$	0.95	10	0.0133	3.0	52.8052
$(S_9Y)_{15}$	0.95	1	0.0734	17.0	48.0658
$(S_9Y)_{15}$	0.95	5	0.0906	9.5	48.0658
$(S_9Y)_{15}$	0.95	10	0.0295	6.0	48.0658
$(S_{12}Y_3)_{10}$	0.95	1	0.0438	16.5	45.8733
$(S_{12}Y_3)_{10}$	0.95	5	0.0793	9.0	45.8733
$(S_{12}Y_3)_{10}$	0.95	10	0.1414	4.0	45.8733
$(S_{24}Y_6)_5$	0.95	1	0.1861	13.0	43.9439
$(S_{24}Y_6)_5$	0.95	5	0.1470	10.0	43.9439
$(S_{24}Y_6)_5$	0.95	10	0.1270	9.0	43.9439
$S_{120}Y_{30}$	0.95	1	0.0323	27.3	56.5004
$S_{120}Y_{30}$	0.95	5	0.0234	19.0	56.5004
$S_{120}Y_{30}$	0.95	10	0.0652	12.0	56.5004
$(S_{120}Y_{30})^{scr}$	0.95	1	0.0582	14.5	44.7216
$(S_{120}Y_{30})^{scr}$	0.95	5	0.0926	7.7	44.7216
$(S_{120}Y_{30})^{scr}$	0.95	10	0.0941	4.0	44.7216
$(SSY)_{50}$	0.95	1	0.0342	18.0	54.5406
$(SSY)_{50}$	0.95	5	0.0661	8.2	54.5406
$(SSY)_{50}$	0.95	10	0.0228	4.0	54.5406
$(YS)_{75}$	0.95	1	0.0293	17.5	50.4438
$(YS)_{75}$	0.95	5	0.0623	7.5	50.4438
$(YS)_{75}$	0.95	10	0.0122	2.7	50.4438
Y_{150}	0.95	1	0.0352	18.6	51.9253
Y_{150}	0.95	5	0.0640	8.0	51.9253
Y_{150}	0.95	10	0.0279	3.6	51.9253
$(YYS)_{50}$	0.95	1	0.0544	22.0	59.4124
$(YYS)_{50}$	0.95	5	0.0682	8.0	59.4124
$(YYS)_{50}$	0.95	10	0.0150	3.2	59.4124

TABLE S3. Maximal measurement values for node betweenness centrality C_B (unitless), node degree (unitless), and single-chain radius of gyration R_g (Å) from simulations of YS variant sequences at $T = 0.95T_c$. Tabulated values are used to normalize measurements of C_B, degree, and R_g in visualizing their relationship to molecular distance from the condensate center of mass in Fig. S18.

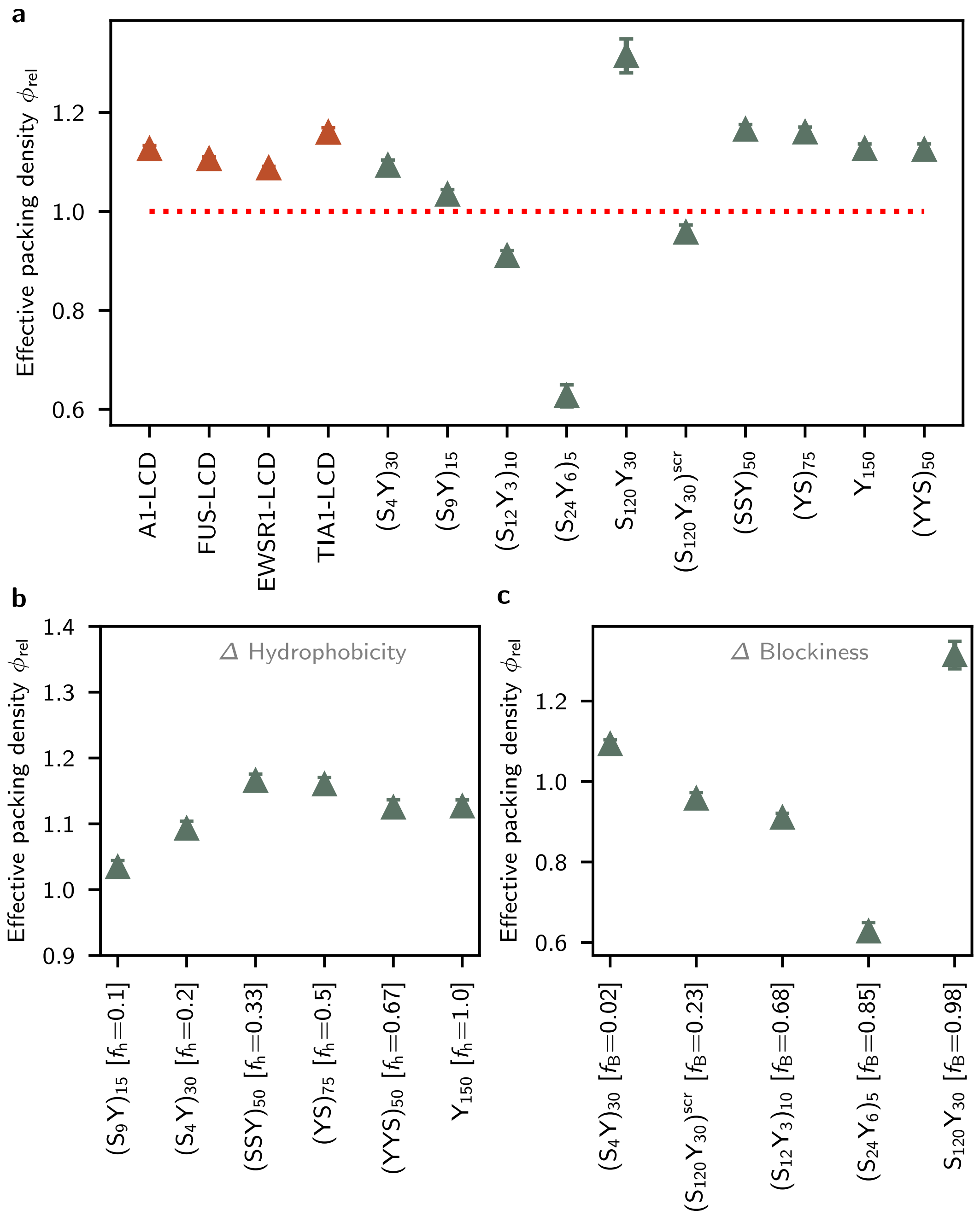

FIG. S19. **a** The effective packing density ϕ_{rel} of chains within all condensates simulated at $T = 0.90\,T_c$. Effective packing densities, or dimensionless densities, are computed with respect to the overlap concentration $c*$ derived from the excluded volume of a dilute single chain (described in the Methods). $\phi_{rel} < 1$ indicates that the condensate dense phase is dilute-like, where individual chains do not overlap; $\phi_{rel} \approx 1$ indicates that the chains in the dense phase are just beginning to overlap, and that polymer overlap or interpenetration is beginning to affect macroscopic qualities of the condensate; $\phi_{rel} > 1$ indicates that the chains in the dense phase are substantially overlapping, and that the macroscopic qualities of the condensate are strongly influenced by polymer interpenetration. **b** The effective packing densities of condensates formed by YS sequences with varying hydrophobicities, simulated at $T = 0.90\,T_c$. **c** The effective packing densities of condensates formed by YS sequences with varying sequence blockiness, simulated at $T = 0.90\,T_c$.
